# PKA activity is essential for relieving the suppression of hyphal growth and appressorium formation by MoSfl1 in *Magnaporthe oryzae*

**DOI:** 10.1371/journal.pgen.1006954

**Published:** 2017-08-14

**Authors:** Yang Li, Xue Zhang, Shuai Hu, Huiquan Liu, Jin-Rong Xu

**Affiliations:** 1 Purdue-NWAFU Joint Research Center, College of Plant Protection, Northwest A&F University, Yangling, Shaanxi, China; 2 Department of Botany and Plant Pathology, Purdue University, West Lafayette, Indiana, United States of America; Oregon State University, UNITED STATES

## Abstract

In the rice blast fungus *Magnaporthe oryzae*, the cAMP-PKA pathway regulates surface recognition, appressorium turgor generation, and invasive growth. However, deletion of *CPKA* failed to block appressorium formation and responses to exogenous cAMP. In this study, we generated and characterized the *cpk2* and *cpkA cpk2* mutants and spontaneous suppressors of *cpkA cpk2* in *M*. *oryzae*. Our results demonstrate that *CPKA* and *CPK2* have specific and overlapping functions, and PKA activity is essential for appressorium formation and plant infection. Unlike the single mutants, the *cpkA cpk2* mutant was significantly reduced in growth and rarely produced conidia. It failed to form appressoria although the intracellular cAMP level and phosphorylation of Pmk1 MAP kinase were increased. The double mutant also was defective in plant penetration and Mps1 activation. Interestingly, it often produced fast-growing spontaneous suppressors that formed appressoria but were still non-pathogenic. Two suppressor strains of *cpkA cpk2* had deletion and insertion mutations in the *MoSFL1* transcription factor gene. Deletion of *MoSFL1* or its C-terminal 93-aa (*MoSFL1*^ΔCT^) was confirmed to suppress the defects of *cpkA cpk2* in hyphal growth but not appressorium formation or pathogenesis. We also isolated 30 spontaneous suppressors of the *cpkA cpk2* mutant in *Fusarium graminearum* and identified mutations in 29 of them in *FgSFL1*. Affinity purification and co-IP assays showed that this C-terminal region of MoSfl1 was essential for its interaction with the conserved Cyc8-Tup1 transcriptional co-repressor, which was reduced by cAMP treatment. Furthermore, the S211D mutation at the conserved PKA-phosphorylation site in *MoSFL1* partially suppressed the defects of *cpkA cpk2*. Overall, our results indicate that PKA activity is essential for appressorium formation and proper activation of Pmk1 or Mps1 in *M*. *oryzae*, and phosphorylation of MoSfl1 by PKA relieves its interaction with the Cyc8-Tup1 co-repressor and suppression of genes important for hyphal growth.

## Author summary

The cAMP-PKA signaling pathway plays a critical role in regulating various cellular processes in eukaryotic cells in response to extracellular cues. In the rice blast fungus, this important pathway is involved in surface recognition, appressorium morphogenesis, and infection. However, the exact role of PKA is not clear due to the functional redundancy of two PKA catalytic subunits *CPKA* and *CPK2*. To further characterize their functions in growth and pathogenesis, in this study we generated and characterized the *cpkA cpk2* double mutant and its suppressor strains. Unlike the single mutants, *cpkA cpk2* mutant had severe defects in growth and conidiation and was defective in appressorium formation and plant infection. Interestingly, the double mutant was unstable and produced fast-growing suppressors. In two suppressor strains, mutations were identified in a transcription factor gene orthologous to *SFL1*, a downstream target of PKA in yeast. Deletion of the entire or C-terminal 93 residues of *MoSFL1* could suppress the growth defect of *cpkA cpk2*. Furthermore, the terminal region of MoSfl1 was found to be essential for its interaction with the MoCyc8 co-repressor, which may be negatively regulated by PKA. Therefore, loss-of-function mutations in *MoSFL1* can bypass PKA activity to suppress the growth defect of *cpkA cpk2*.

## Introduction

*Magnaporthe oryzae* is the causal agent of rice blast, which is one of the most important rice diseases worldwide. In the past two decades, *M*. *oryzae* has been developed as a model organism to study fungal-plant interactions because of its economic importance and the experimental tractability [1–3]. For plant infection, the fungus forms a highly specialized infection cell called an appressorium to penetrate plant cuticle and cell wall [4]. After penetration, the narrow penetration peg differentiates into bulbous invasive hyphae [5] that grow biotrophically inside penetrated plant cells [6]. Various apoplastic and cytoplasmic effectors are known to play critical roles in suppressing plant defense responses during different stages of invasive growth [7]. At late infection stages, lesions are formed and the pathogen produces conidiophores and conidia on diseased plant tissues under favorable conditions.

Appressorium formation is initiated when conidia land and germinate on plant surfaces. On artificial hydrophobic surfaces that mimic the rice leaf surface, *M*. *oryzae* also forms melanized appressoria. On hydrophilic surfaces, appressorium formation can be induced by cAMP, IBMX, or cutin monomers [8]. Although late stages of appressorium formation is regulated by the Pmk1 MAP kinase, the cAMP-PKA (protein kinase A) pathway is involved in recognizing surface hydrophobicity to initiate appressorium formation, appressorium turgor generation, and invasive growth [9–11]. Deletion of the *MAC1* adenylate cyclase (AC) gene results in mutants that are defective in appressorium formation [12]. In addition to Cap1 AC-interacting protein [13], heterotrimeric G-proteins and Rgs1 have been shown to function upstream from the cAMP-PKA pathway [2, 14]. The PdeH high-affinity cAMP phosphodiesterase is also important for successful establishment and spread of the blast disease [15].

The PKA holoenzyme consists of two regulatory subunits and two catalytic subunits. Binding of cAMP with the regulatory subunit results in the detachment and activation of the catalytic subunits [16]. In *M*. *oryzae*, the *CPKA* gene encoding a catalytic subunit of PKA is dispensable for hyphal growth but the *cpkA* mutant was delayed in appressorium formation and defective in appressorium turgor generation and plant penetration. In addition, the *cpkA* mutant still responds to exogenous cAMP for appressorium formation on hydrophilic surfaces [10, 11], suggesting that another PKA catalytic subunit gene must exist and play a role in surface recognition and infection-related morphogenesis in *M*. *oryzae*. In the budding yeast *Saccharomyces cerevisiae*, three genes, *TPK1*, *TPK2*, and *TPK3*, encode PKA catalytic subunits and the triple mutant is inviable [17]. The fission yeast *Schizosaccharomyces pombe* has only one PKA catalytic subunit gene, *PKA1*, that is important but not essential for normal growth [18]. In the human pathogen *Aspergillus fumigatus*, the *pkaC1 pkaC2* double mutant is delayed in conidium germination in response to environmental nutrients and is significantly reduced in virulence [19]. In the wheat scab fungus *Fusarium graminearum*, deletion of both *CPK1* and *CPK2* caused severe defects in growth and conidiation. The *cpk1 cpk2* double mutant was sterile in sexual reproduction and nonpathogenic [20]. In the basidiomycete *Ustilago maydis*, the phenotype of the *adr1 uka1* double mutant has similar phenotype with the *adr1* mutant and is defective in yeast growth, mating, and plant infection [21].

In *S*. *cerevisiae*, Sfl1 is one of the downstream transcription factors of the cAMP-PKA pathway. When functioning as a repressor, it is involved in the repression of flocculation-related genes, including *FLO11* and *SUC2* [22, 23]. As an activator, *SFL1* is involved in the activation of stress-responsive genes such as *HSP30* [24]. The major PKA catalytic subunit Tpk2 negatively regulates its repressor function [25]. In *M*. *oryzae*, deletion of *MoSFL1* has no obvious effect on vegetative growth but results in reduced virulence and heat tolerance [26]. Several Sfl1-interacting proteins have been identified in the budding yeast, including Cyc8, Tup1, and various mediator components [23, 27]. Although it lacks intrinsic DNA-binding activities, the Cyc8-Tup1 (also known as Ssn6-Tup1) co-repressor complex interacts with various transcription factors with sequence-specific DNA binding motifs, including Sfl1, Mig1, Crt1, and α2, to negatively regulate different subsets of genes [27, 28]. In *S*. *cerevisiae*, Cyc8 functions as an adaptor protein required for the interaction between Tup1 tetramers and DNA-binding transcription factors [29].

To further characterize the roles of PKA in growth and infection, in this study we generated and characterized the *cpk2* and *cpkA cpk2* double mutants and spontaneous suppressors of *cpkA cpk2* in *M*. *oryzae*. Our results demonstrate that *CPKA* and *CPK2* have specific and overlapping functions. The *cpkA cpk2* double mutant had severe defects in growth and conidiation and failed to form appressoria or infect plant through wounds. Spontaneous mutations or deletion and truncation mutations in *MoSFL1* suppressed the defects of *cpkA cpk2* in hyphal growth and appressorium formation but not invasive growth and lesion development. In affinity purification and co-IP assays, MoCyc8 interacted with the full-length but not truncated MoSfl1. Treatment with exogenous cAMP also reduced the interaction of MoSfl1 with MoCyc8 and MoTup1. Furthermore, the S211D mutation in *MoSFL1* suppressed the growth defect of *cpkA cpk2*. In *F*. *graminearum*, 29 of 30 suppressor strains of *cpk1 cpk2* mutant had mutations in *FgSFL1*, with 15 of them truncated of its C-terminal region. Taken together, our results indicate that PKA activity is essential for appressorium formation in *M*. *oryzae*, and phosphorylation of MoSfl1 by PKA likely relieves its interaction with the Cyc8-Tup1 co-repressor and suppression of genes important for hyphal growth and appressorium development. The inhibitory function of Sfl1 orthologs on hyphal growth is likely conserved in filamentous fungi because similar suppressor mutations in *FgSFL1* were identified in spontaneous suppressor strains of the *cpk1 cpk2* mutant in *F*. *graminearum*.

## Results

### Both CpkA and Cpk2 interact with the PKA regulatory subunit Sum1

In *M*. *oryzae*, the PKA regulatory subunit is encoded by *SUM1*, a suppressor of the *mac1* deletion mutant [30]. In an effort to identify proteins interacting with known virulence factors, including Sum1, we generated the *SUM1*-S-tag fusion and transformed it into the wild-type strain 70–15. Total proteins were isolated from the resulting transformant B22 (Table 1) and subjected to affinity purification and MS analysis after trypsin digestion as described [13, 31]. CpkA and Cpk2 (MGG_02832) were among the Sum1-interacting proteins identified in all three independent biological replicates (S1 Table). Cpk2 shares 48% amino acid identity with CpkA but it has a shorter N-terminal region (S1 Fig).

**Table 1:** Wild-type and mutant strains of *Magnaporthe oryzae* used in this study.

Strain	Genotype description	Reference
Guy11	Wild type (*MAT1*-2)	[32]
nn78	*pmk1* mutant of Guy11	[9]
M3H51	*mps1* mutant of Guy11	[33]
DF51	*cpkA* deletion mutant of Guy11	[10]
YP18	*cpk2* deletion mutant	This study
CAC2	*cpkA cpk2* deletion mutant	This study
B22	*SUM1*-S transformant of 70–15	This study
KAS5	*SUM1*-S and *CPKA*-3×Flag transformant of Guy11	This study
KZS2	*SUM1*-S and *CPK2*-3×Flag transformant of Guy11	This study
CCS1 to CCS20	Spontaneous suppressor mutants of the *cpkA cpk2* mutant of *M*. *oryzae*	This study
CTD2	*cpkA cpk2 MoSFL1*^ΔCT^ mutant	This study
TKO4	*cpkA cpk2 Mosfl1* triple mutant	This study
SFL2	3×Flag-*MoSFL1* transformant of *cpkA cpk2* mutant CAC2	This study
SCT7	3×Flag-*MoSFL1*^**Δ**CT^ transformant of *cpkA cpk2* mutant CAC2	This study
CYS15	*MoCYC8*-S and 3×Flag-*MoSFL1* transformant of CAC2	This study
CNC19	*MoCYC8*-S and 3×Flag-*MoSFL1*^**Δ**CT^ transformant of CAC2	This study
GCS1	*MoCYC8*-S and 3×Flag-*MoSFL1* transformant of Guy11	This study
GTS9	*MoTUP1*-S and 3×Flag-*MoSFL1* transformant of Guy11	This study
MTU35	*Motup1* deletion mutant	This study
ASD5	*MoSFL1*^S211D^ transformant of *cpkA cpk2* mutant CAC2	This study
HTD17	*MoSFL1*^T441D^ transformant of *cpkA cpk2* mutant CAC2	This study
GSD22	*MoSFL1*^S554D^ transformant of *cpkA cpk2* mutant CAC2	This study
ASA9	*MoSFL1*^S211A^ transformant of *cpkA cpk2* mutant CAC2	This study
HTA3	*MoSFL1*^T441A^ transformant of *cpkA cpk2* mutant CAC2	This study
GSA6	*MoSFL1*^S554A^ transformant of *cpkA cpk2* mutant CAC2	This study
HS1 to HS30	Spontaneous suppressor mutants of the *cpk1 cpk2* mutant of *F*. *graminearum*	This study

To confirm their interaction by co-immunoprecipitation (co-IP) assays, the *SUM1*-S, *CPKA*-3×FLAG, and *CPK2*-3×FLAG constructs were generated and transformed into the wild-type strain Guy11 in pairs. Western blot analysis with the resulting transformants showed that both PKA catalytic subunits strongly interact with Sum1 (S2 Fig).

### The *cpkA cpk2* double mutant has severe growth and conidiation defects

**Fig 1:**
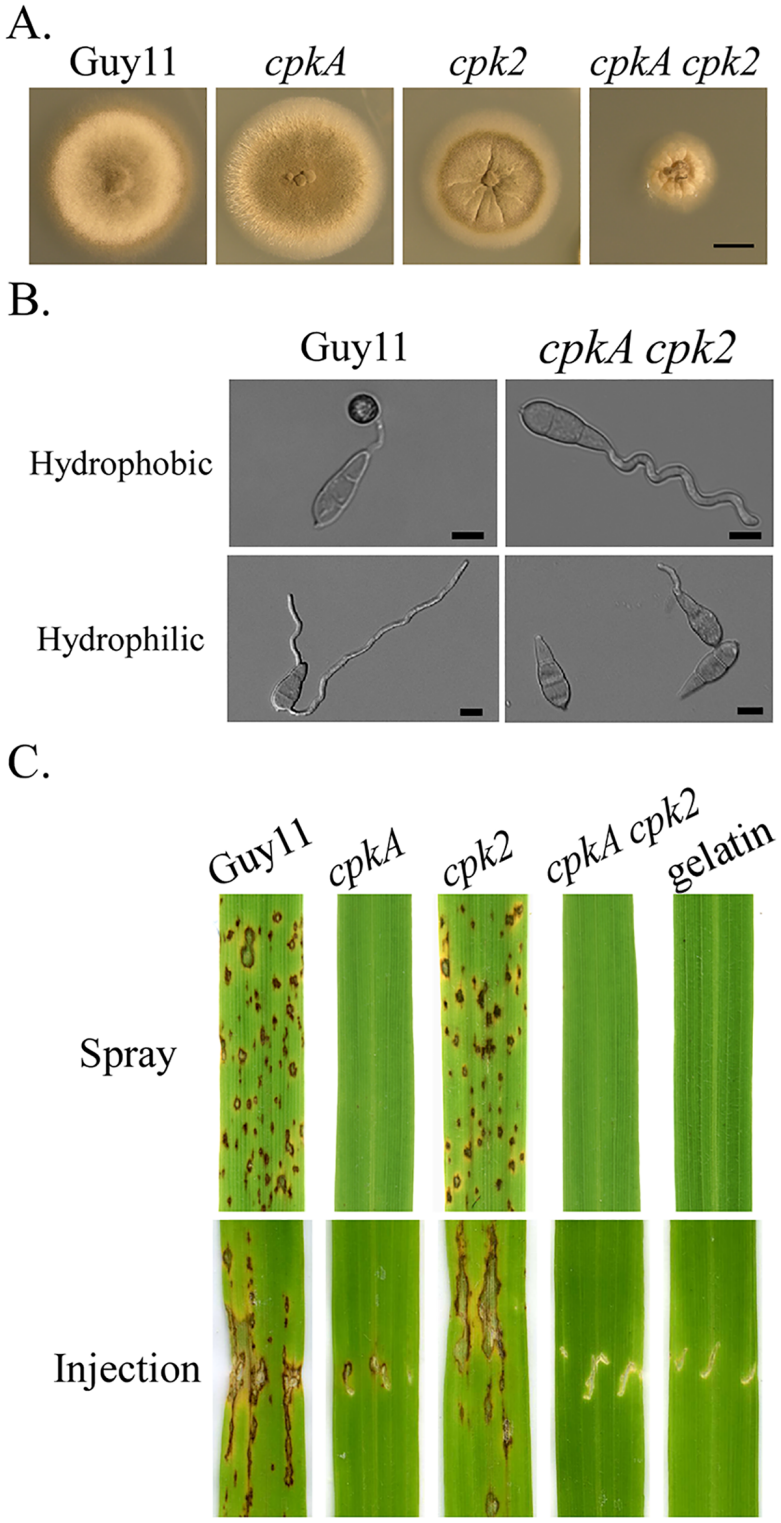
Defects of the *cpkA cpk2* mutant in growth, appressorium formation, and plant infection. **A**. Seven-day-old cultures of the wild-type Guy11 and *cpkA*, *cpk2*, and *cpkA cpk2* mutants. Scale bar = 1 cm. **B**. Conidia of Guy11 and mutant strains were incubated on the hydrophobic (upper panel) and hydrophilic (lower panel) surface of GelBond membranes for 24 h. Scale bar = 10 μm. **C**. Leaves of two-week-old rice seedlings were inoculated by spray or injection with conidium suspensions of labelled strains. Inoculation with 0.25% gelatin was used as the negative control. Typical leaves were photographed 7 dpi.

To determine the function of PKA catalytic subunits, we generated the *cpk2* and *cpkA cpk2* deletion mutants (Table 1; S1 Fig). On complete medium (CM) plates, the *cpkA* mutant had no obvious growth defects but the *cpk2* mutant was slightly reduced in growth rate. The *cpkA cpk2* double mutant was viable but it was significantly reduced in growth rate (Table 2; Fig 1A). Unlike *cpkA* and *cpk2*, the *cpkA cpk2* double mutant rarely produced conidia. In cultures induced for conidiation, the double mutant produced only a few conidia per plate. Under the same conditions, over 1×10^7^ conidia/plate were produced by the wild type (Table 2).

**Table 2:** Growth rate, conidiation and appressoria formation of the wild type and the *cpkA*, *cpk2*, and *cpkA cpk2* mutants.

Strain	Growth rate (mm/day) ^a^	Conidiation (×10^5^ conidia/plate)	Appressoria formation (%)^b^
Guy11 (wt)	3.0±0.0 ^A^	88.7±8.8 ^A^	96.7±2.5 ^A^
DF51 (*cpkA*)	2.9±0.1 ^A^	92.2±6.7 ^A^	95.6±2.5 ^A^
YP18 (*cpk2*)	2.5±0.1 ^B^	84.8±5.8 ^A^	97.3±2.1 ^A^
CAC2 (*cpkA cpk2*)	1.4±0.1 ^C^	Rare	0 ^B^

^**a**^ Growth rate and conidiation were assayed with 7- and 14-days-old OTA cultures, respectively.

^**b**^ Percentage of germ tubes that formed appressoria on the hydrophobic side of GelBond membranes. Means and standard errors were estimated with results from three independent experiments. Data were analyzed with Duncan's pair wise comparison.

Different letters mark statistically significant differences (P = 0.05).

### PKA activities are essential for appressorium formation and plant infection

Unlike *cpkA* that was delayed in appressorium formation, *cpk2* had no obvious defects in appressorium formation (Table 2). However, the *cpkA cpk2* mutant failed to form appressoria on hydrophobic plastic coverslips and GelBond membranes although conidium germination was normal (Fig 1B; Table 2). Even after prolonged incubation up to 72 h, no appressorium formation was observed in the double mutant. The *cpkA cpk2* mutant also failed to form appressoria on barley and rice leaves (S3 Fig).

In spray infection assays with two-week-old seedlings of rice cultivar CO-39, numerous blast lesions were observed on leaves sprayed with Guy11 or the *cpk2* mutant but no lesions were caused by the *cpkA* and *cpkA cpk2* mutants (Fig 1C). Because the c*pkA cpk2* mutant failed to form appressoria, we conducted injection infection assays. Whereas the *cpk2* mutant was as virulent as the wild type, the *cpkA* mutant failed to cause lesions on intact leaves but caused limited necrosis at the wounding sites. No lesions or necrosis at the wounding sites were observed on leaves inoculated with the *cpkA cpk2* mutant (Fig 1C). These results indicate that PKA activities are essential for appressorium formation and invasive growth after penetration in *M*. *oryzae*.

### The *cpkA cpk2* mutant still recognizes surface hydrophobicity

Although the *cpkA cpk2* mutant was blocked in appressorium formation, we noticed that majority (over 83%) of its germ tubes were curved one direction after incubation on hydrophobic side of GelBond membranes for 24 h (Fig 1B). Interestingly, when assayed for appressorium formation on the hydrophilic side of GelBond membranes, the majority of the *cpkA cpk2* conidia failed to germinate (Fig 1B; Table 3). For the ones (<25%) germinated, germ tubes of *cpkA cpk2* mutant failed to form appressoria (Fig 1B). Conidia of the wild-type, *cpkA*, and *cpk2* strains germinated but failed to form appressoria under the same conditions. In the presence of 5 mM cAMP, over 76% of the wild-type germ tubes formed appressoria on hydrophilic surfaces. However, exogenous cAMP had no stimulatory effects on either conidium germination or appressorium formation in the *cpkA cpk2* mutant (Fig 1B; Table 3). Over 75% of the double mutant conidia failed to germinate and the ones germinated failed to form appressoria or display germ tube curling defects in the presence of 5 mM cAMP. These results indicate that the *cpkA cpk2* mutant still recognizes surface hydrophobicity for germination and germ tube growth but not for appressorium formation.

**Table 3:** Conidium germination and appressorium formation on hydrophilic surfaces.

Strain	Germination (%)		Appressorium formation (%)	
	No cAMP	5 mM cAMP *	No cAMP	5 mM cAMP *
Guy11 (WT)	99.7±0.6 ^A^	99.0±1.0 ^A^	0.0±0.0	76.3±6.7
CAC2 (*cpkA cpk2*)	23.0±7.2 ^B^	28.3±5.0 ^B^	0.0±0.0	0.0±0.0

* Percentage of conidia germinated after incubation on the hydrophilic (with or without 5 mM cAMP) or hydrophobic side of GelBond membranes for 24 h. Means and standard errors were calculated with results from three independent experiments, with at least 100 conidia examined per replicate. Data were analyzed with Duncan's pair wise comparison.

Different letters mark statistically significant differences (P = 0.05).

### Elevated intracellular cAMP levels in the *cpkA* and *cpkA cpk2* mutants

**Fig 2:**
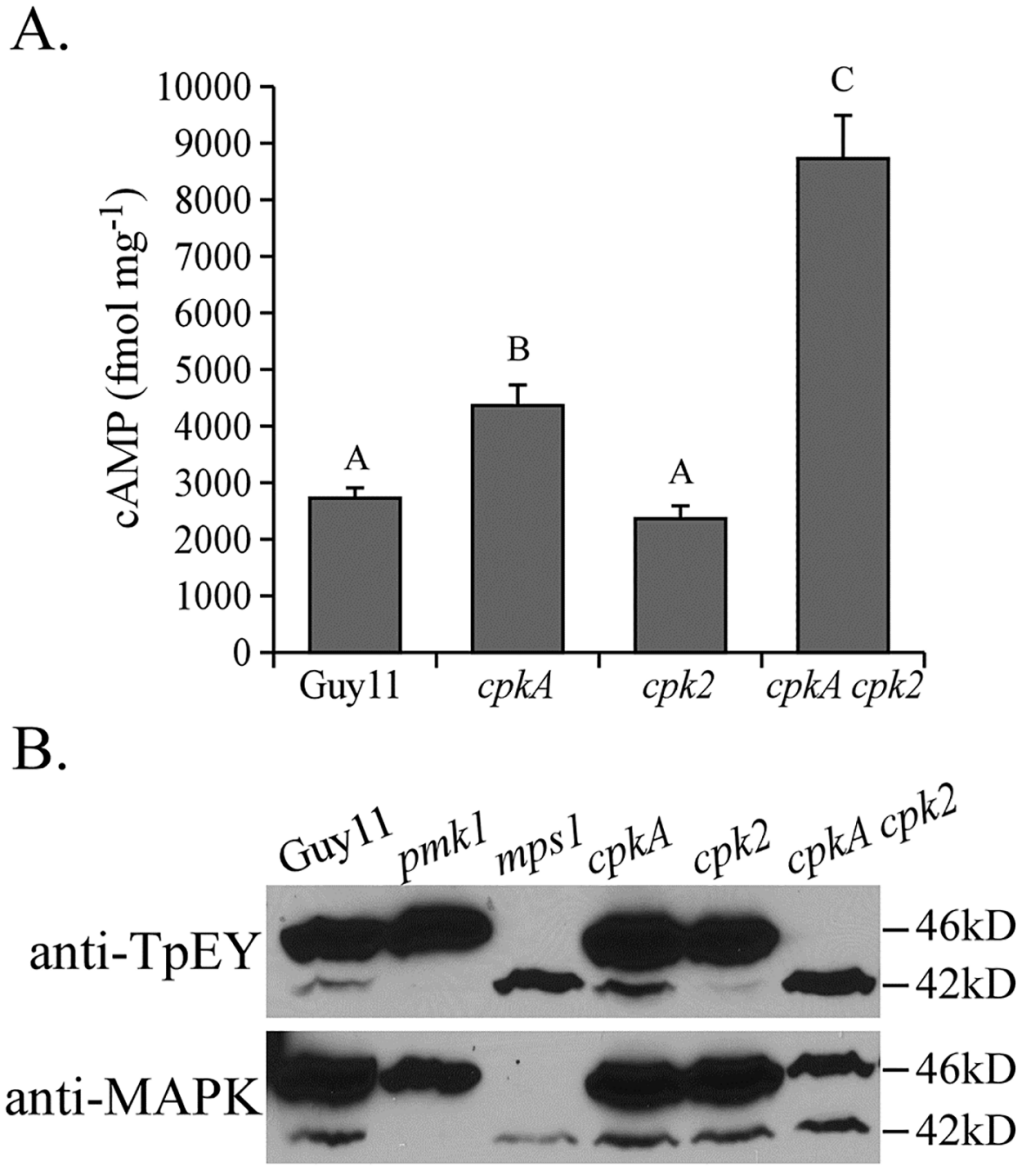
Assays for the intracellular cAMP level and activation of Pmk1 or Mps1 MAP kinase. **A**. The intracellular cAMP level was assayed with vegetative hyphae of Guy11 and the *cpkA*, *cpk2*, and *cpkA cpk2* mutants. Mean and standard deviation were calculated with results from three independent biological replicates. Different letters mark statistically significant differences (P = 0.05). **B**. Western blots of proteins isolated from Guy11 and the *pmk1*, *mps1*, *cpkA*, *cpk2*, and *cpkA cpk2* mutant were detected with an anti-TpEY specific (upper panel) or anti-MAPK (lower panel) antibody to assay the phosphorylation of Pmk1 (42-kD) and Mps1 (46-kD).

We then assayed the intracellular cAMP level in vegetative hyphae harvested from liquid CM cultures. In the *cpk2* mutant, the intracellular cAMP level was similar to that of the wild type. However, the *cpkA* and *cpkA cpk2* mutants had higher intracellular cAMP levels than the wild type (Fig 2A). In comparison with the wild type, the double mutant was increased approximately 3-fold in intracellular cAMP. These results suggest that reduced or lack of PKA activities results in an increase in intracellular cAMP in *M*. *oryzae*.

### Absence of PKA activities interferes with the Pmk1 and Mps1 MAP kinase pathways

Because the Pmk1 MAP kinase is essential for appressorium formation [34], we assayed its activation with an anti-TpEY phosphorylation specific antibody. To our surprise, although its expression was not affected, Pmk1 phosphorylation was increased in the *cpkA cpk2* mutant (Fig 2B). However, the double mutant was reduced in the expression and phosphorylation levels of Mps1 MAP kinase (Fig 2B) that is required for appressorium penetration and conidiation [33]. These results suggest that over-activation of the Pmk1 MAP kinase pathway is not sufficient to stimulate appressorium formation in the absence of PKA activities, and reduced Mps1 activities may be related to conidiation defects of *cpkA cpk2*.

### Spontaneous suppressors of *cpkA cpk2* are partially recovered in growth but not pathogenesis

**Fig 3:**
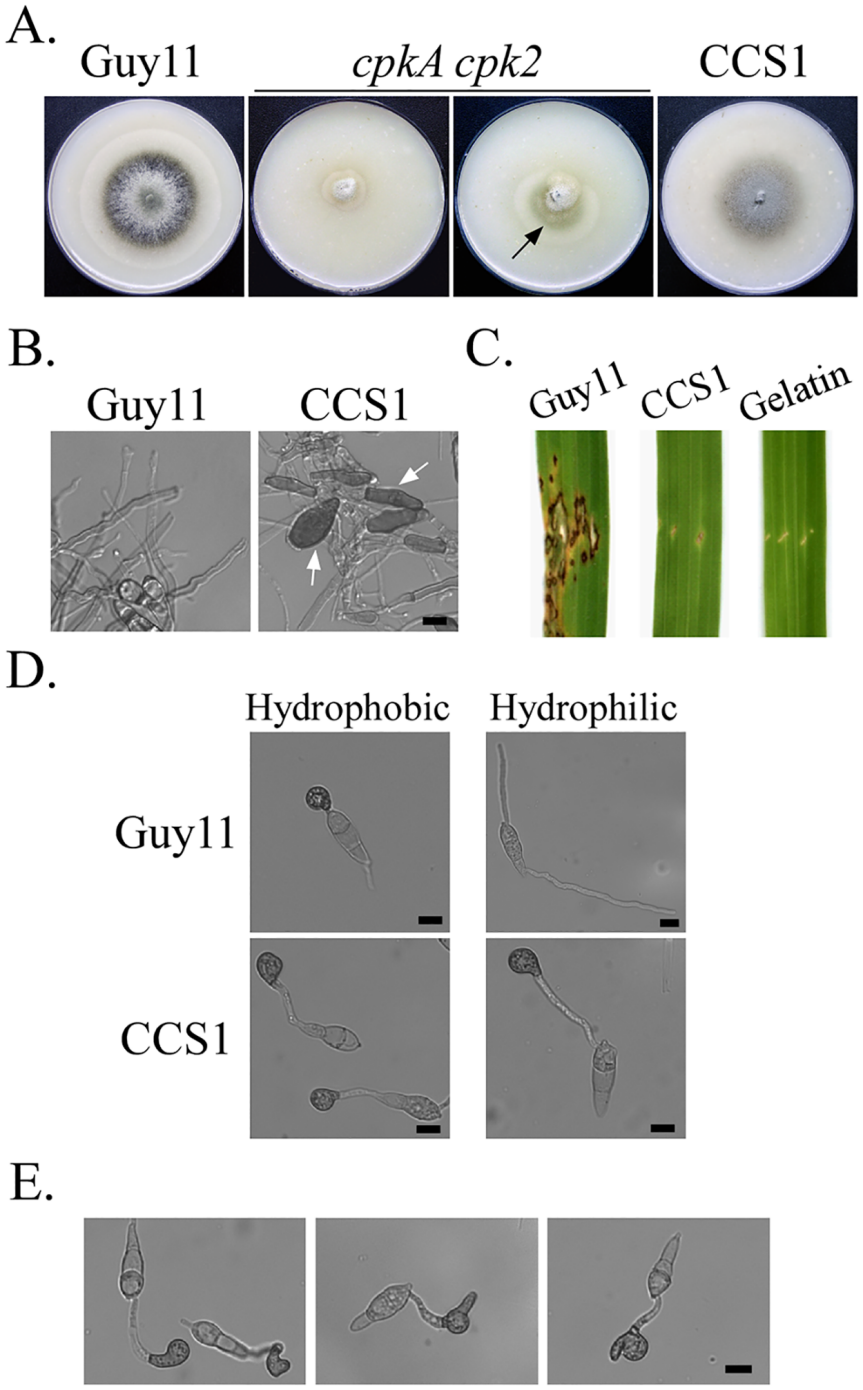
Phenotypes of the spontaneous suppressor strain CCS1 of *cpkA cpk2*. **A**. Seven-day-old OTA cultures of the wild type strain Guy11, *cpkA cpk2* mutant, and suppressor strain CCS1. The fast-growing suppressor of *cpkA cpk2* was marked with an arrow. **B**. Hyphal tips of Guy11 and CCS1 from 7-day-old OTA cultures. Arrows point to melanized hyphal tips. Scale bar = 10 μm. **C**. Rice leaves inoculated by injection with conidia of Guy11 and CCS1 were examined 7 days-post-inoculation. **D**. Appressoria formed by Guy11 and CCS1 on the hydrophobic and hydrophilic surfaces after incubation for 24 h. Scale bar = 10 μm. **E**. Abnormal appressoria formed by CCS1 on the hydrophobic surface after incubation for 24 h. Scale bar = 10 μm.

Interestingly, the *cpkA cpk2* mutant was unstable when cultured on the oatmeal agar (OTA) plates and fast-growing sectors caused by spontaneous suppressor mutations often became visible in cultures older than 10 days (Fig 3A). Twenty suppressor strains with faster growth rate than the original mutant were isolated. All of them had similar colony morphology and produced more aerial hyphae than the *cpkA cpk2* mutant. On average, the growth rate of suppressor strains recovered to approximately 83% of that of the wild type (S4 Fig). Conidiation also was partially rescued in these suppressor strains although to a much lesser degree than the recovery in growth rate (S4 Fig).

Although they varied slightly in growth rate and conidiation (S4 Fig), all the 20 suppressor strains had similar defects as strain CCS1 that was described and presented in figures below. Besides having similar colony morphology, suppressor strains produced melanized hyphal tips in aerial hyphae of 10-day-old OTA cultures (Fig 3B). In infection assays with rice seedlings, none of the suppressor strains caused lesions on intact or wounded leaves (Fig 3C). Therefore, mutations occurred in these suppressor strains only suppressed the defects of the *cpkA cpk2* mutant in hyphal growth but not plant infection.

### Appressoria are formed by the suppressor strains of *cpkA cpk2*

On artificial hydrophobic surfaces, over 95% of the conidia from the suppressor strains formed appressoria after incubation for 24 h. Interestingly, they also developed appressoria on the hydrophilic surface of GelBond membranes (Fig 3D). However, approximately 40% of appressoria formed by suppressor strains were abnormal in morphology on hydrophobic or hydrophilic surfaces (Fig 3E). Unlike normal dome-shaped appressoria, the majority of appressoria formed by suppressor strains had irregular shapes (Fig 3E). Although they were still melanized, many appressoria formed by the suppressor strains had projections at one side (Fig 3E). These results indicate that suppressor mutations in these strains also only partially rescued the defect of *cpkA cpk2* in appressorium morphogenesis.

### Identification of suppressor mutations in *MoSFL1*

To identify suppressor mutations, we selected eight genes (S2 Table) [27, 35–40] that are orthologous to downstream targets of PKA in the budding yeast, including *SOM1* and *CDTF1* [40] for PCR and sequencing analysis in the selected suppressor strains CCS1, CCS4, CCS7 and CCS14. Whereas suppressor stains CCS4 and CCS14 had no mutations in these candidate genes, both CCS1 and CCS7 had mutations in the *MoSFL1* [26] gene that encodes a transcription factor with a conserved HSF (heat shock factor) DNA-binding domain in the N-terminal region (residues 124–225) [26]. In suppressor strain CCS1, 10 extra nucleotides CCCCCGCCGC were inserted in the coding region of *MoSFL1* (between 1556 and 1557), resulting in a frameshift change at residue P414. In suppressor strain CCS7, a 1241-bp deletion occurred in the coding region of *MoSFL1* (Δ405–1645), resulting in the truncation of 78% of its amino acids.

### Deletion of *MoSFL1* rescues the defects of the *cpkA cpk2* mutant

**Fig 4:**
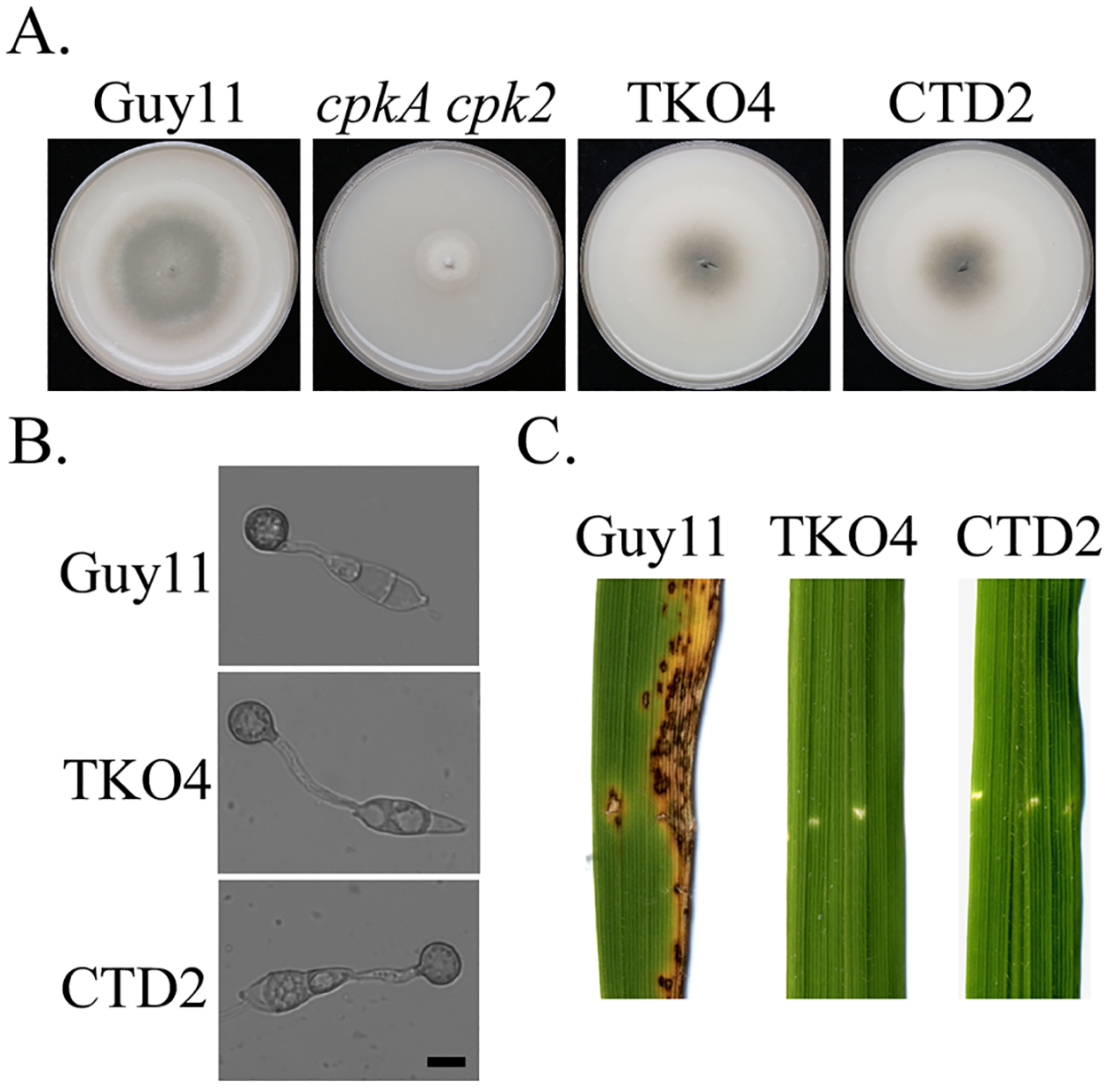
Deletion of *MoSFL1* and *MoSFL*^CT^ in the *cpkA cpk2* mutant. **A**. Seven-day-old oatmeal agar plates of Guy11, *cpkA cpk2* mutant, *cpkA cpk2 Mosfl1* (TKO4), and *cpkA cpk2 MoSFL1*^ΔCT^ (CTD2) triple mutants. **B**. Appressorium formation assays with Guy11, TKO4, and CTD2 strains on the hydrophobic surface of GelBond membranes for 24 h. Scale bar = 10 μm. **C**. Leaves of two-week-old rice seedlings were injected with conidium suspensions of Guy11, TKO4, and CTD2 strains. Typical leaves were photographed 7 dpi.

In *M*. *oryzae*, deletion of *MoSFL1* had no effects on hyphal growth although it was reduced in virulence [26]. To confirm whether insertion or truncation mutation in *MoSFL1* has suppressive effects, the *MoSFL1* gene replacement construct was transformed into the *cpkA cpk2* mutant. Bleomycin-resistant transformants were screened by PCR for deletion of *MoSFL1* and confirmed by Southern blot (S5 Fig). The resulting *cpkA cpk2 Mosfl1* mutant had similar phenotypes as spontaneous suppressor strains (Fig 4; Table 4), including recovered growth rate and increased conidiation in comparison with *cpkA cpk2*. Melanized appressoria were efficiently formed by the triple mutant but it failed to cause lesions on rice leaves, further confirming that loss-of-function mutations in *MoSFL1* rescue the growth defect of *cpkA cpk2*. Therefore, *MoSFL1* must function as a negative regulator of vegetative hyphal growth and phosphorylation of MoSfl1 by PKA relieves its suppressive effects.

**Table 4:** Growth rate and conidiation of the *cpkA cpk2 Mosfl1* and *cpkA cpk2 MoSFL*^ΔCT^ mutants.

Strain	Growth rate (mm/day)	Conidiation (×10^5^ conidia/plate)
Guy11 (WT)	3.4±0.0 ^A^	165.5±24.4 ^A^
CAC2 (*cpkA cpk2*)	0.9±0.1 ^B^	Rare
TKO4 (*cpkA cpk2 Mosfl1*)	3.0±0.0 ^A^	1.7±0.5 ^B^
CTD2 (*cpkA cpk2 MoSFL*^ΔCT^)	3.1±0.1 ^A^	1.5±0.3 ^B^

Means and standard errors were estimated with results from three independent measurements. Data were analyzed with Duncan's pair wise comparison.

Different letters mark statistically significant differences (P = 0.05).

### The C-terminal region is important for *MoSFL1* function as a negative regulator

In suppressor strain CCS1, the 10-bp insertion in *MoSFL1* causes frameshift and results in the truncation of its C-terminal 414–588 aa. Sequence alignment showed that this C-terminal region of MoSfl1 is well conserved among its orthologs from other filamentous fungi, including *Fusarium graminearum* and *Neurospora crassa* (S6 Fig). To verify its importance, we generated a *MoSFL1*^ΔCT^ gene-replacement construct (S5 Fig) to delete residues 496–588 of *MoSFL1* in the *cpkA cpk2* mutant. The resulting *cpkA cpk2 MoSFL1*^ΔCT^ triple mutants (S5 Fig) had the same phenotypes with the *cpkA cpk2 Mosfl1* mutant and spontaneous suppressor strains (Fig 4; Table 4). These results suggested that the C-terminal region of MoSfl1 is essential for its negative regulator function although it has no known protein motifs. Whereas the N-terminal region of MoSfl1 is involved in DNA binding, the C-terminal region may be responsible for protein-protein interactions to suppress the expression of its target genes important for hyphal growth.

### Identification of proteins differentially interacting with MoSfl1 and MoSfl1^ΔCT^

Because deletion of residues 496–588 is suppressive to *cpkA cpk2*, this C-terminal region of MoSfl1 is likely responsible for interacting with other proteins as a negative regulator. To identify proteins differentially interacting with MoSfl1 and MoSfl1^ΔCT^, the 3×FLAG-*MoSFL1* and 3×FLAG-*MoSFL1*^**Δ**CT^ constructs were generated and transformed into the *cpkA cpk2* mutant. Total proteins were isolated from the resulting 3×FLAG-*MoSFL1* and 3×FLAG-*MoSFL1*^**Δ**CT^ transformants and used for affinity purification with anti-FLAG M2 beads. Proteins co-purified with MoSfl1 or MoSfl1^**Δ**CT^ were identified by mass spectrometry (MS) analysis after trypsin digestion as described in previous studies [13, 31].

Based on MS results from three biological replicates, MGG_03196 was the only protein that co-purified with MoSfl1 but not MoSfl1^**Δ**CT^ (Table 5). Its ortholog in yeast, Cyc8 (Ssn6), forms a transcriptional co-repressor complex with Tup1 to regulate genes involved in a wide variety of physiological processes [28, 41, 42]. Interestingly, the Tup1 ortholog, MGG_08829, was one of the proteins that were commonly co-purified with MoSfl1 and MoSfl1^**Δ**CT^ (Table 5). However, the number of MoTup1 peptides identified by MS analysis was significantly lower in the *MoSFL1*^**Δ**CT^ transformant than in the *MoSFL1* transformant (Table 5), suggesting a weaker interaction of MoSfl1 with MoTup1 when its C-terminal region is deleted. Based on the conserved nature of Tup1, Cyc8, and other components, it is likely that the Cyc8 and Tup1 orthologs also form a transcriptional co-repressor complex with MoSfl1 in *M*. *oryzae*, which is consistent with the interaction of Sfl1 with the Cyc8-Tup1 complex in yeast [27].

**Table 5:** Putative *MoSFL1*- and *MoSFL1*^ΔCT^-interacting proteins identified by affinity purification.

Genes	Annotation	% PSMs of *	
		*MoSFL1*	*MoSFL1*^ΔCT^
MGG_06958	Hsp70-like protein	63	65
MGG_04191	Hsp70-like protein	36	38
MGG_02503	Glucose-regulated protein	17	16
MGG_03286	Pathway-specific nitrogen regulator	9	10
MGG_13806	14-3-3 family protein	8	12
MGG_01842	Uncharacterized protein	3	3
MGG_01720	Transcription regulatory protein Swi3	11	8
**MGG_08829**	**Transcriptional repressor Tup1**	**26**	**4**
MGG_01268	Nuclear localization sequence binding protein	3	4
MGG_01588	14-3-3 family protein	3	3
MGG_09565	Mitogen-activated protein kinase Pmk1	2	3
**MGG_03196**	**Cyc8**	**10**	**0**

* PSMs (peptide spectrum matches): The average number of identified peptide sequences for the protein from three biological replicates. PSMs values of *MoSFL1* and *MoSFL1*^ΔCT^ were normalized to 100.

### The C-terminal region of MoSfl1 is important for its interaction with Cyc8

**Fig 5:**
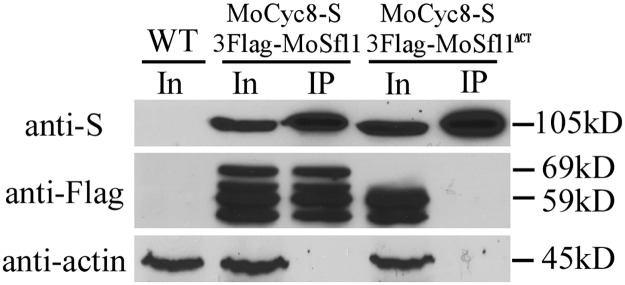
Co-IP assays for the interaction of MoCyc8 with MoSfl1 and MoSfl1^ΔCT^. Western blots of total proteins (In for input) isolated from transformants expressing the *MoCYC8*-S and 3×Flag-*MoSFL1* (CYS15) or 3×Flag-MoSfl1^ΔCT^ (CNC19) constructs and proteins immuno-precipitated (IP) with anti-S-Tag agarose beads were detected with the anti-S and anti-Flag antibodies. Detection with an anti-actin antibody was included as the negative co-IP control. The expected sizes of MoCyc8-S, 3×Flag-MoSfl1, and 3×Flag-MoSfl1^ΔCT^ were labelled on the right.

To confirm the importance of the C-terminal region of MoSfl1 in its interaction with Cyc8, the *CYC8-*S-tag construct was generated and co-transformed into the *cpkA cpk2* mutant with 3×FLAG-*MoSFL1* or -*MoSFL1*^**Δ**CT^. The resulting transformants CYS15 and CNC19 (Table 1) were confirmed by western blot analyses for the expression of transforming constructs. In co-IP assays, the MoSfl1 band was detected in both total proteins and elution from anti-S-tag agarose beads in the transformant expressing the *CYC8*-S and 3×FLAG-*MoSFL1* constructs (Fig 5). However, the MoSfl1^**Δ**CT^ band was detected only in total proteins isolated from the transformant expressing *CYC8*-S and 3×FLAG-*MoSFL1*^**Δ**CT^ (Fig 5). These results confirmed that Cyc8 interacts with the full-length MoSfl1 but not MoSfl1^**Δ**CT^ in *M*. *oryzae*. Interestingly, additional bands smaller than MoSfl1 or MoSfl1^**Δ**CT^ were detected by the anti-FLAG antibody in transformants CYS15 and CNC19 but not in Guy11, suggesting that MoSfl1 proteins may be cleaved in vegetative hyphae.

### Exogenous cAMP reduces the interaction of MoSfl1 with MoCyc8

**Fig 6:**
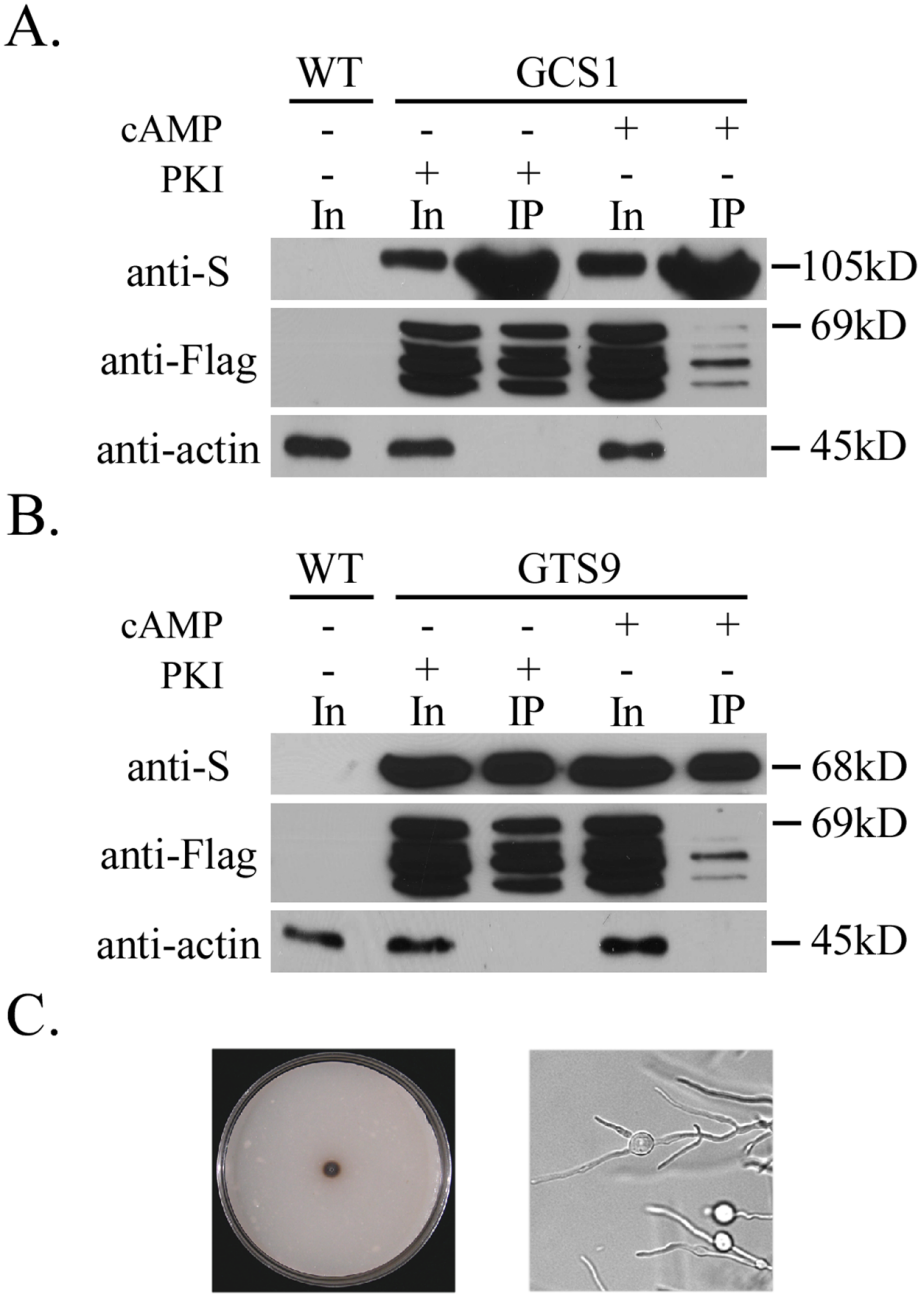
Assays for the effects of cAMP or PKA inhibitor H-89 on the MoSfl1-MoCyc8 interaction. **A**. Western blots of total proteins (In for input) and proteins immuno-precipitated (IP) with anti-S-Tag agarose beads were detected with an anti-S, anti-Flag, or anti-actin antibody. Proteins were isolated from transformant GCS1 expressing *MoCYC8*-S and 3×Flag-*MoSFL1* constructs cultured under labelled treatments (+) or not (−). **B**. Western blots of total proteins and proteins immuno-precipitated with anti-S-Tag agarose beads of transformant GTS9 expressing *MoTUP1*-S and 3×Flag-*MoSFL1* constructs were detected with an anti-S, anti-Flag, or anti-actin antibody. Total proteins isolated from the wild-type strain Guy11 (WT) were included as the control. **C**. Seven-day-old CM cultures of the *Motup1* mutant had limited growth and apical or subapical swollen bodies in hyphae.

Because deletion of the C-terminal region of *MoSFL1* suppressed the growth defect of *cpkA cpk2*, phosphorylation of MoSfl1 by PKA may affect its interaction with the Cyc8-Tup1 complex. To test this hypothesis, the *MoCYC8*-S and 3×FLAG-*MoSFL1* constructs were co-transformed into the wild-type strain Guy11. In the resulting transformant, treatment with 5 mM cAMP significantly reduced the MoCyc8-MoSfl1 interaction compared to treatment with 10 μM PKA inhibitor (PKI) H-89 (Fig 6A). These results indicate that stimulation of PKA activities by exogenous cAMP reduces the interaction of MoSfl1 with MoCyc8. Therefore, phosphorylation of MoSfl1 by PKA likely reduced the interaction of MoSfl1 with the co-repressor MoCyc8 to negative regulation of hyphal growth-related genes.

### *MoTUP1* also interacts with MoSfl1 and is important for hyphal growth and pathogenesis

We also generated transformants of Guy11 expressing the *MoTUP1*-S and 3×FLAG-*MoSFL1* constructs. MoTup1 was found to interact with MoSfl1 in co-IP assays with the resulting transformant (Fig 6B). Treatment with exogenous cAMP also significantly reduced the interaction of MoSfl1 with MoTup1 (Fig 6B). These results indicate that stimulation of PKA activities by exogenous cAMP reduces the interaction of MoSfl1 with MoCyc8 and MoTup1.

To determine the function of *MoTUP1*, we generated the *Motup1* deletion mutant in the wild-type strain Guy11. The resulting deletion mutant MTU35 (Table 1) was significantly reduced in growth rate and it rarely produced aerial hyphae and conidia (Fig 6C). Unlike the *cpkA cpk2* mutant, the *Motup1* mutant was normal in appressorium formation. One distinct phenotype of the *Motup1* mutant was the production of swollen bodies in the subapical regions in hyphae grown on CM (Fig 6C), suggesting cell wall integrity defects. The phenotype differences between the *Motup1* mutant and *Mosfl1* or *cpkA cpk2* mutant indicate that the MoCyc8-MoTup1 co-repressor is involved in regulating different sets of genes by interacting with transcription factors other than MoSfl1.

### Phosphorylation of S211 in MoSfl1 rescues the growth defect of *cpkA cpk2*

**Fig 7:**
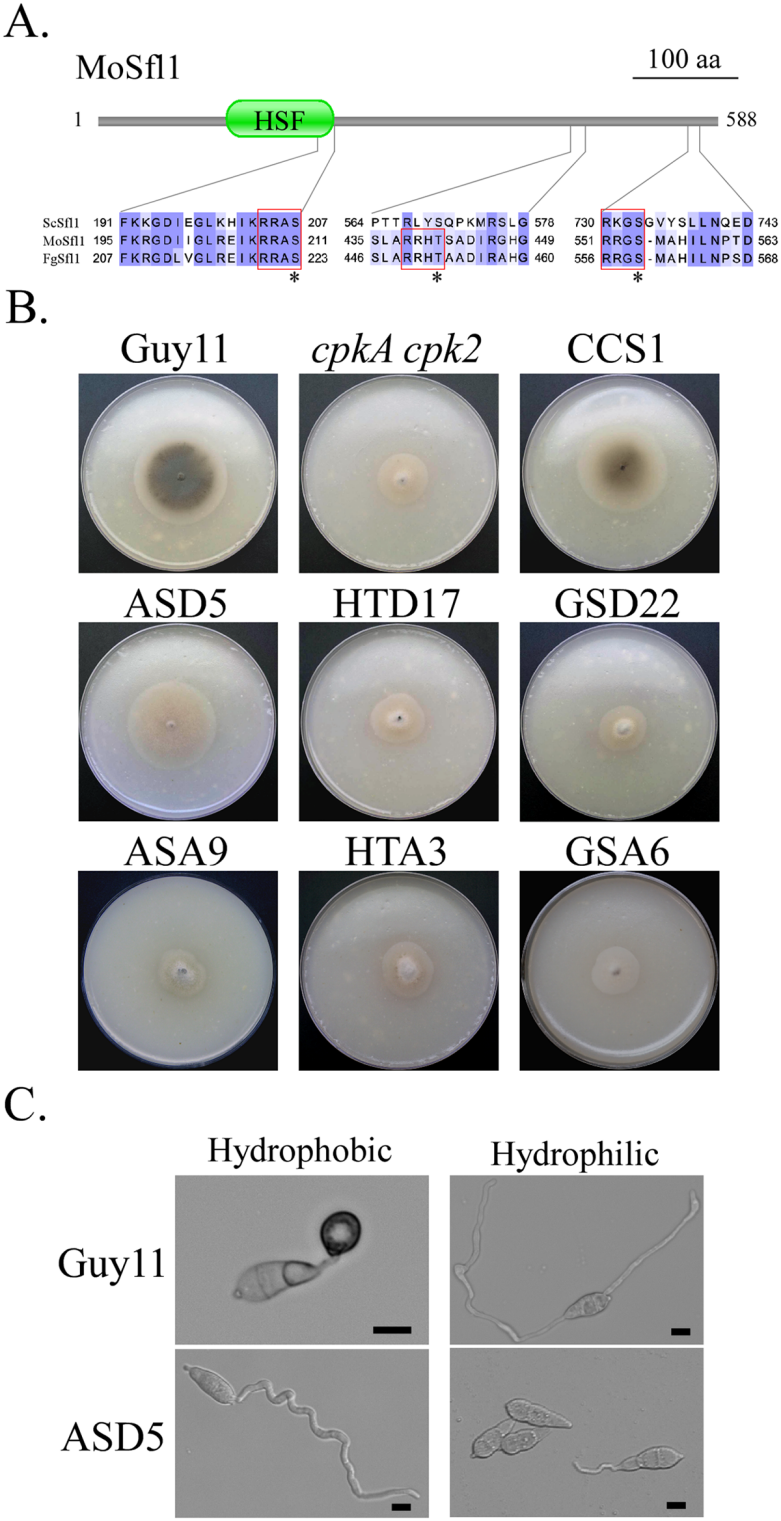
Site-directed mutagenesis of putative PKA phosphorylation sites in MoSfl1. **A**. Schematic drawing of the MoSfl1 protein and alignment of the marked region with its orthologs from *F*. *graminearum* (Fg) and *S*. *cerevisiae* (Sc). The consensus PKA phosphorylation sites were boxed with red lines. The putative PKA phosphorylation residues were marked with stars. **B**. Five-day-old OTA cultures of the wild-type strain Guy11, *cpkA cpk2* mutant, and transformants of *cpkA cpk2* expressing the *MoSFL1*^S211D^ (ASD5), *MoSFL1*^S211A^ (ASA9), *MoSFL1*^T441D^ (HTD17), *MoSFL1*^T441A^ (HTA17), *MoSFL1*^S554D^ (GSD22), or *MoSFL1*^S554A^ (GSA22) allele. **C**. Conidia of Guy11 and *MoSFL1*^S211D^ transformant ASD5 were assayed for appressorium formation on the hydrophobic side of GelBond membranes.

In *S*. *cerevisiae*, Sfl1 has two predicted consensus PKA phosphorylation sites S207 and S733 [43] that are conserved in MoSfl1 (S211 and S554) and its orthologs from other fungi (Fig 7A). PSITE analysis identified T441 as the only other consensus PKA phosphorylation site in MoSfl1. To determine their functions, the *MoSFL1*^S211D^, *MoSFL1*^T441D^, and *MoSFL1*^S554D^ alleles were generated and transformed into the *cpkA cpk2* mutant. Whereas *MoSFL1*^S211D^ transformants grew faster, the *MoSFL1*^T441D^ and *MoSFL1*^S554D^ transformants had similar growth defects with the original *cpkA cpk2* mutant (Fig 7B). Similar approaches were used to generate transformants of *cpkA cpk2* expressing the *MoSFL1*^S211A^, *MoSFL1*^T441A^, and *MoSFL1*^S554A^ alleles (Table 1). None of these S/T to A mutations had suppressive effects on the growth defect of *cpkA cpk2* (Fig 7B). These results indicate that phosphorylation of MoSfl1 at S211 may play a critical role to release its inhibitory functions. However, the *MoSFL1*^S211D^ transformant failed to form appressoria on hydrophobic surfaces (Fig 7C). Therefore, the S211D mutation in *MoSFL1* could suppress the growth but not appressorium formation defect of *cpkA cpk2*.

### Genes down-regulated in the *cpkA cpk2* mutant are enriched for the putative Sfl1-binding site in their promoters

To identify genes affected by deletion of both *CPKA* and *CPK2*, we conducted RNA-seq analysis with RNA isolated from hyphae collected from 2-day-old CM cultures. Considering the significant reduction in growth rate, it was surprising that only 451 genes were down-regulated in the *cpkA cpk2* double mutant in comparison with the wild type (S3 Table). However, many of them are functionally important for growth, including several genes encoding ribosomal proteins (MGG_00546, MGG_03372, MGG_09927, MGG_01113, MGG_06571) and enzymes important for cell wall synthesis (MGG_00592, MGG_03208, MGG_07331, MGG_01575, and MGG_03883). When the promoter regions (1000-bp upstream of the start codon) of genes down-regulated in the double mutant were analyzed, 111 of them contain the putative HSF-binding element AGAA-n-TTCT (n≤20) [27]. Among them, 29 genes have more than one HSF-binding elements in their promoter regions. These results indicate that the putative MoSfl1-binding element is enriched among the genes significantly down-regulated in the double mutant.

### Spontaneous suppressors of *cpk1 cpk2* and mutations in *FgSFL1* in *F*. *graminearum*

**Fig 8:**
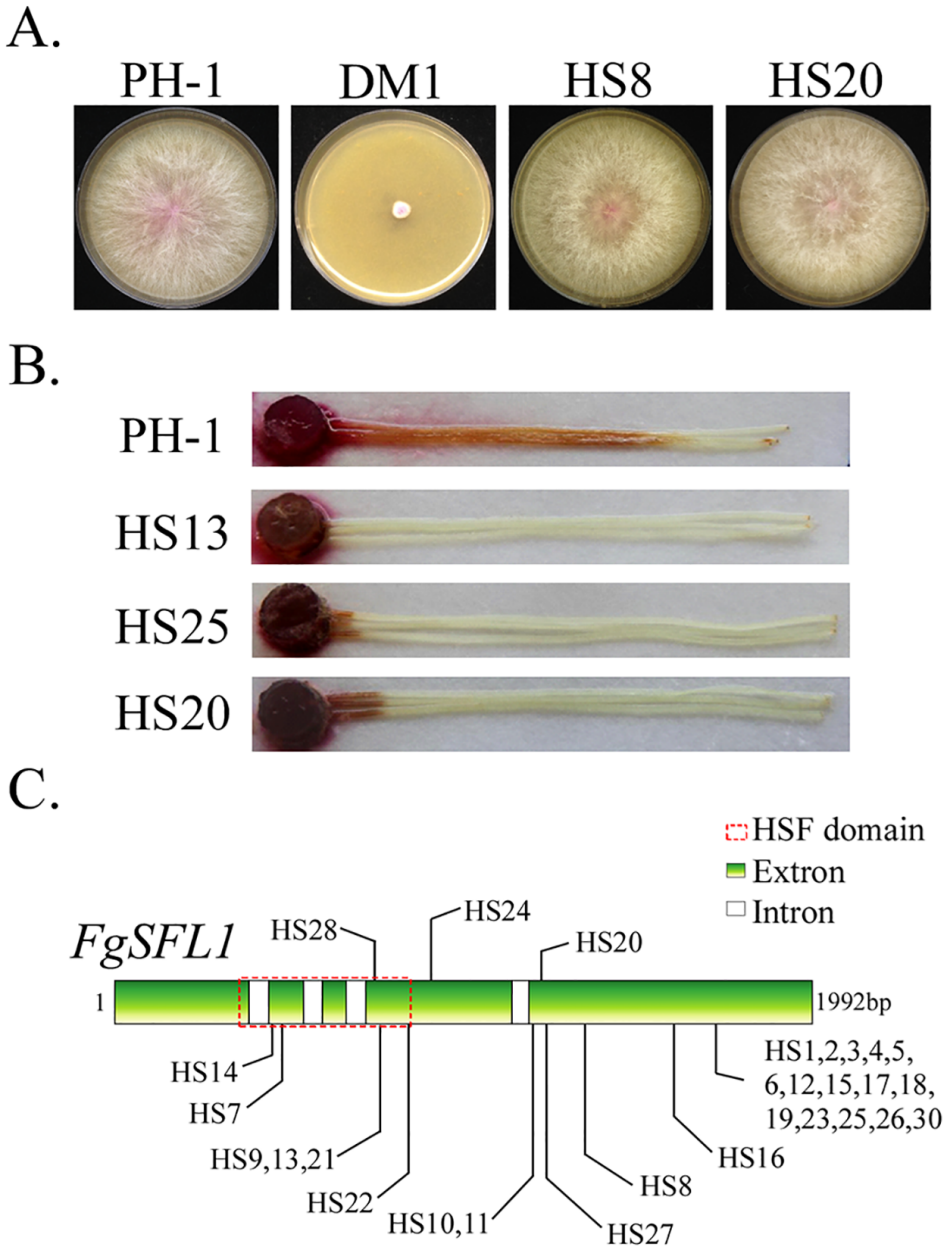
Spontaneous suppressors of the *cpk1 cpk2* mutant in *F*. *graminearum*. **A**. Four-day-old V8 cultures of the wild-type strain PH-1, *cpk1 cpk2* mutant (DM1), and suppressor strains HS8 and HS20. **B**. Corn silks inoculated with culture blocks of suppressor strains HS13, HS25, and HS20. **C**. Suppressor mutations identified in *FgSFL1*.

The *cpk1 cpk2* double mutant of *F*. *graminearum* also had severe growth defects [20]. Similar to the *cpkA cpk2* mutant of *M*. *oryzae*, fast-growing sectors were often observed in V8 cultures of *cpk1 cpk2* mutant that were older than 10 days. We isolated 30 suppressor strains that had similar growth rate with the wild type (Fig 8A). In infection assays with corn silks, suppressor strains of *cpk1 cpk2* were still defective in plant infection (Fig 8B). When the *FgSFL1* gene was amplified and sequenced, 29 of them had mutations in the open reading frame (ORF) (Fig 8C; S4 Table). Suppressor strain HS29 lacked mutations in the ORF of *FgSFL1* although its phenotype was similar to that of other suppressor strains with mutations in *FgSFL1*, suggesting possible mutations at its interacting site on *FgCYC8*. The most common mutation is the C1717 to T (Q501 to stop codon) mutation that resulted in the truncation of C-terminal 91 amino acids. A total of 15 suppressor strains had this C1717T mutation. Therefore, truncation of the C-terminal region also suppressed the *cpk1 cpk2* mutant in *F*. *graminearum*. These results indicate that the function of *SFL1* orthologs in hyphal growth is well conserved in *M*. *oryzae*, *F*. *graminearum*, and possibly other filamentous ascomycetes.

## Discussion

Like many other filamentous ascomycetes, the rice blast fungus has two genes encoding catalytic subunits of PKA. Whereas the *cpkA* mutant is defective in appressorium formation and pathogenesis, deletion of *CPK2* had no effects on plant infection and appressorium morphogenesis. Interestingly, unlike *cpkA*, the *cpk2* mutant was slightly reduced in growth rate. Therefore, although CpkA plays a more critical role than Cpk2 for pathogenesis, *CPK2* may be more important during vegetative growth. In *F*. *graminearum* and *A*. *fumigatus*, deletion of the *CPK2* ortholog had no detectable phenotype [19, 20], which differs slightly from *cpk2* in *M*. *oryzae*. However, the *cpkA cpk2* mutant had more severe defects than the single mutants in growth and conidiation, which is similar to *F*. *graminearum* and *A*. *fumigatus* [19, 20]. Therefore, the overlapping functions of CpkA and Cpk2 orthologs during vegetative growth and asexual reproduction may be evolutionally conserved in filamentous ascomycetes.

Although cAMP signaling is known to be important for appressorium formation in a number of fungal pathogens, including *Colletotrichum* species [44, 45], no mutants deleted of both catalytic subunits have been reported in plant pathogenic fungi except in *U*. *maydis*. In *U*. *maydis*, the mutant deleted of both catalytic subunits was defective in plant infection but its defects in appressorium formation was not examined [21]. Our results showed that PKA activities are essential for appressorium formation in *M*. *oryzae*, which has not been previous reported in plant pathogens.

The cAMP-PKA pathway is responsible for surface recognition in *M*. *oryzae*. To our surprise, although no tip swelling or appressorium formation was observed, the majority of *cpkA cpk2* germ tubes curled or rotated on hydrophobic surfaces. Therefore, germ tubes of the double mutant still responded to surface hydrophobicity although they were blocked in appressorium formation. Sensing of surface hardness and hydrophobicity likely involves mechanosensor proteins, which may trigger polarity disturbance and germ tube curling independent of cAMP signaling. Several putative mechanosensor genes have been shown to be up-regulated during appressorium formation [46]. It is puzzling that most *cpkA cpk2* conidia failed to germinate on hydrophilic surfaces, and curling germ tubes were not observed in a few of them that germinated. In filamentous ascomycetes such as *A*. *nidulans* and *C*. *trifolii*, cAMP signaling is known to regulate conidium germination [44, 47]. However, the *cpkA cpk2* mutant was normal in conidium germination on hydrophobic surfaces. Because surface attachment is a cue for stimulating conidium germination in *M*. *oryzae*, one likely explanation is that deletion of both *CPKA* and *CPK2* may affect the attachment of conidia to hydrophilic surfaces.

The Pmk1 MAP kinase pathway is essential for appressorium formation in *M*. *oryzae* and other plant pathogens [9, 34]. The *cpkA cpk2* mutant had an increased phosphorylation level of Pmk1 but was defective in appressorium formation. It is possible that PKA activity is required to release the suppressive effect of MoSfl1 on genes important for germ tube tip swelling and appressorium formation. Spontaneous suppressors of *cpkA cpk2* produced melanized tips in aerial hyphae and appressoria on hydrophilic surfaces, which is similar to transformants expressing the dominant active *RAS2* [48]. Therefore, releasing the repressor role of MoSfl1 or MoCyc8-MoTup1 in the *cpkA cpk2* mutant in which Pmk1 is over-activated is sufficient to activate appressorium formation under non-conducive conditions. One likely explanation is that some genes required for tip deformation or appressorium formation are only expressed when MoSfl1 is phosphorylated by PKA although the essential role of Pmk1 in appressorium formation involve other downstream targets. Because deletion of *MoSFL1* had no effect on appressorium formation [26], it will be interesting to determine the effects of expressing the dominant active *MST7* allele in the *Mosfl1* deletion mutant.

In *M*. *oryzae*, the *mac1* mutant is known to produce spontaneous suppressors and some of them had suppressor mutations in the *SUM1* gene [30]. In fact, instability of adenylate cyclase mutant and suppressor mutations in regulatory subunit genes are well characterized in *S*. *cerevisiae*, *U*. *maydis*, and other fungi [30, 49, 50]. However, to our knowledge, spontaneous suppressors of PKA mutants have not been reported in other fungi. In *S*. *cerevisiae*, the *tpk1 tpk2 tpk3* triple mutant is not viable. It will be important to assay whether the mutants deleted of both catalytic subunits also produce spontaneous suppressors in *U*. *maydis* and *A*. *fumigatus* [19, 21]. Because the *cpk1 cpk2* mutant of *F*. *graminearum* [20] was also found to be unstable and had mutations in *FgSFL1* in 29 of the 30 suppressor strains sequenced, it is likely that *SFL1* orthologs have a conserved role in the repression of genes important for growth and conidiation, at least in filamentous ascomycetes. For the one *F*. *graminearum* and two *M*. *oryzae* suppressor strains without mutations in the *SFL1* ortholog, identification of the suppressor mutations by whole genome sequencing [51] and characterization of the corresponding genes in these mutants in the future will be helpful to better understand the cAMP-PKA pathway in filamentous fungi.

In yeast, *SFL1* can function as either a transcriptional activator or repressor [23, 24]. In *M*. *oryzae*, deletion of *MoSFL1* by itself did not affect vegetative growth but resulted in a reduction in virulence [26]. Phenotype characterization of the *Mosfl1* mutant is suitable for characterizing its activator but not repressor functions. In this study, we showed that deletion or truncation of *MoSFL1* could suppress the defects of *cpkA cpk2* mutant in vegetative growth and appressorium formation but not plant infection and conidiation. Interestingly, the suppressor mutants with mutations in *FgSFL1* were also recovered in vegetative growth but not pathogenicity in *F*. *graminearum*. Whereas *M*. *oryzae* forms melanized appressoria for plant penetration, *F*. *graminearum* produces infection cushions and hyphopodia [52, 53]. However, after plant penetration, both of them form invasive hyphae inside plant cells that are different from vegetative hyphae in hyphal morphology and possibly cell cycle regulation [52, 54, 55]. The cAMP-PKA pathway is important for plant infection in *M*. *oryzae* [34, 56] and *F*. *graminearum* [20, 53], possibly by regulating the growth of invasive hyphae after penetration in these two plant pathogenic fungi with different infection mechanisms. It is possible that *MoSFL1* plays an activator role in regulating genes important for invasive growth but negatively regulates genes important for vegetative growth in *M*. *oryzae*. However, it is more likely that the cAMP-PKA pathway regulates genes important for plant penetration and invasive growth via other transcription factors.

**Fig 9:**
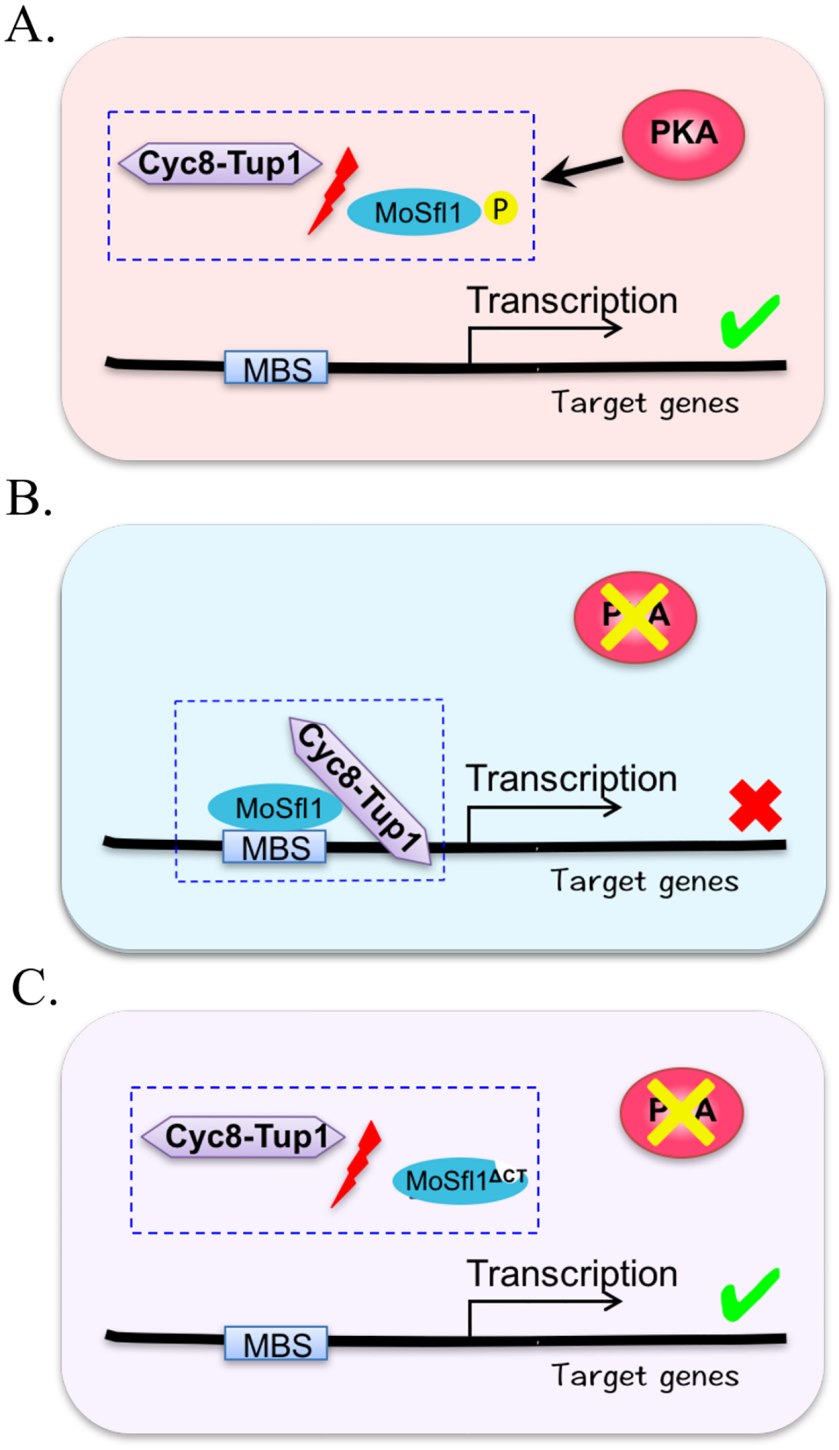
A model of the repressive role of MoSfl1 and its phosphorylation by PKA in transcriptional regulation. **A**. In the wild-type strain, phosphorylation of MoSfl1 by PKA disrupts its interaction with the Cyc8-Tup1 co-repressor. Without its association with MoSfl1, the Cyc8-Tup1 complex fails to block the transcription of MoSfl1 target genes that are important for hyphal growth and other developmental or infection processes. **B**. In the *cpkA cpk2* mutant, the Cyc8-Tup1 co-repressor interacts with un-phosphorylated MoSfl1 to repress subsets of its target genes important for growth. **C**. Suppressor mutations in *MoSFL1* block or reduce the association of MoSfl1 with the Cyc8-Tup1 co-repressor to bypass the requirement for its phosphorylation by PKA. P, phosphorylation; MBS, MoSfl1-binding site.

In yeast, Sfl1 inhibits the transcription of its target genes by interacting with the Cyc8-Tup1 co-repressor [27]. However, it is not clear which region of Sfl1 interacts with Cyc8 or Tup1. Our data clearly showed that the C-terminal 93 amino acids of MoSfl1 is essential for its interaction with Cyc8 (Fig 9). This C-terminal region of MoSfl1 is well conserved in its orthologs from filamentous ascomycetes. In *F*. *graminearum*, 15 of the 29 suppressor strains of *cpk1 cpk2* mutant had the nonsense mutation at Q501 (S4 Table) in *FgSFL1* resulting in the truncation of its C-terminal region. Other 12 suppressor strains had either nonsense or frameshift mutations upstream from Q501. These results indicate that the C-terminal region of Sfl1 orthologs likely plays a conserved role in regulating the expression of genes important for hyphal growth via its association with the Cyc8-Tup1 co-repressor complex. The difference between MoSfl1 and yeast Sfl1 in the C-terminal region may be directly related to the importance of PKA activities in hyphal growth in *M*. *oryzae*, *F*. *graminearum*, and possibly other filamentous fungi.

Besides the Cyc1-Tup1 co-repressor, Sfl1 also interacts with the mediator proteins Ssn2, Ssn8, Sin4, and Rox3 in *S*. *cerevisiae* [23]. Although their orthologs are conserved in *M*. *oryzae*, none of them was identified by affinity purification. One possibility is that phosphorylation by PKA is necessary for MoSfl1 to interact with these mediator components but the *cpkA cpk2* mutant was used to identify proteins that differentially interacted with MoSfl1 and MoSfl1^ΔCT^ and responsible the suppression of PKA deficiency. Nevertheless, it is also possible that their interactions with MoSfl1 is mediated by the Cyc8-Tup1 complex, which may be too dynamic or transient in *M*. *oryzae*. Among the MoSfl1-interacting proteins identified by affinity purification, MGG_01588 and MGG_06958 are orthologous to *BMH1* and *SSA1*, respectively that interact with Sfl1 in *S*. *cerevisiae*. However, several MoSfl1-interacting proteins in *M*. *oryzae* (Table 5) such as the putative pathway-specific nitrogen regulator MGG_03286 are unique to filamentous fungi. It will be important to determine their functions in hyphal growth and pathogenesis in *M*. *oryzae*.

In *S*. *cerevisiae*, Sfl1 is a substrate of Tpk2 [25, 57]. However, it is not clear whether S207D mutation (= S211 of MoSfl1) is sufficient to suppress the growth defect of inviable *tpk1 tpk2 tpk3* PKA-deficient mutant [58]. In *M*. *oryzae*, expression of the *MoSFL1*^S211D^ allele in the *cpkA cpk2* mutant rescued its growth but not appressorium formation defects. It is possible that phosphorylation of MoSfl1 by PKA disrupts the interaction of MoSfl1 with the Cyc8-Tup1 co-repressor, which in turn activates the expression of genes important for hyphal growth (Fig 9). Residue S211 of MoSfl1 is well conserved in its orthologs from other filamentous ascomycetes, suggesting that its phosphorylation by PKA likely has a conserved role in the regulation of hyphal growth by the cAMP-PKA pathway. Considering the fact that CpkA and Cpk2 are highly similar in the kinase domain and they have overlapping functions in hyphal growth, it is possible that both of them can phosphorylate MoSfl1 at S211. Nevertheless, MoSfl1 may be phosphorylated by CpkA and Cpk2 at different amino acid residues. Therefore, it will be important to characterize the phosphorylation sites of MoSfl1 in the *cpkA* and/or *cpk2* deletion mutants.

## Materials and methods

### Strains and culture conditions

All the *M*. *oryzae* strains used in this study (Table 1) were cultured on oatmeal agar (OTA) or complete medium (CM) plates at 25°C and stored on desiccated Whatman #1 filter paper at -20°C [59]. Protoplast preparation and PEG-mediated transformation were performed as described [60]. Transformants were selected with 250 μg/ml hygromycin B (CalBiochem), 250 μg/ml geneticin G418 (Sigma), or 200 μg/ml zeocin (Invitrogen) in the top agar. Growth rate and conidiation were assayed with OTA cultures as described [60, 61].

### Generation of the *cpk2* and *cpkA cpk2* mutants

To generate the *CPK2* gene replacement construct by double-joint PCR, its 1.2-kb upstream and downstream flanking sequences of *CPK2* were amplified with primer pairs 1F/ 2R and 3F/4R (S5 Table), respectively (S1 Fig). The *hph* cassette was amplified with primers Hyg/F and Hyg/R from pCX63 [62]. The resulting products of double-joint PCR were transformed into protoplasts of the wild-type strain Guy11. Putative *cpk2* mutants were screened by PCR with primers 5F and 6R and further confirmed by Southern blot analyses with its downstream flanking sequence as the probe. Vegetative hyphae harvested from two-day-old CM cultures were used for DNA and protein isolation as described [63].

The same strategy was used to generate the *CPKA* gene replacement construct. The 1.2-kb upstream and downstream flanking sequences of *CPKA* were amplified with primer pairs A1F/A2R and A3F/A4R, respectively (S1 Fig). The G418 cassette was amplified with primers G418/F and G418/R from pFl7. The products of double-joint PCR were transformed into protoplasts of the *cpk2* mutant to generate the *cpkA cpk2* mutant.

### Appressorium formation, penetration, and plant infection assays

Conidia were harvested from 10-day-old OTA cultures and resuspended to 5×10^4^ conidia/ml in sterile water. For appressorium formation assays, 50 μl droplets of conidium suspensions were placed on glass cover slips (Fisher Scientific) or GelBond membranes (Cambrex) and incubated at 25°C for 24 h as described [13, 64]. To assay its stimulatory effects, cAMP was added to the final concentration of 10 μM to conidium suspensions [65]. For infection assays, conidia were resuspended to 5×10^5^ conidia/ml in 0.25% gelatin. Two-week-old seedlings of rice cultivar CO-39 were used for spray or injection infection assays as described [66, 67]. Lesion formation was examined 7 days post inoculation (dpi).

### Assays for the TEY phosphorylation of MAP kinases

Vegetative hyphae were harvested from 2-day-old CM cultures and used for protein extraction as described [68]. Total proteins (approximately 20 mg) were separated on a 12.5% SDS-PAGE gel and transferred to nitrocellulose membranes for western blot analysis [62]. Expression and phosphorylation of Pmk1 and Mps1 were detected with the PhophoPlus p44/42 MAP kinase antibody kits (Cell Signaling Technology) following the manufacturer’s instructions.

### Assays for the intracellular cAMP levels

Intracellular cAMP was extracted from vegetative hyphae harvested from two-day-old CM cultures as described [69] and detected with the cAMP enzyme immunoassay (EIA) system (Amersham Pharmacia Biotech) following the manufacturer’s instructions.

### Co-immunoprecipitation (co-IP) assays

The yeast gap repair approach was used to generate the S-tag and 3×FLAG fusion constructs [68]. To generate the *SUM1-*, *TUP1-*, and *CYC8-*S-tag constructs, each gene was amplified and cloned into vector pXY203 [66, 70]. *MoSFL1* was cloned into vector pFL6 to generate the 3×FLAG-*MoSFL1* construct. *CPKA* and *CPK2* were cloned into vector pFL7 [70] to generate the *CPKA*-3xFLAG and *CPK2*-3×FLAG fusion constructs. All of the resulting S-tag and 3×FLAG fusion constructs were confirmed by sequencing analysis and transformed into Guy11 or the *cpkA cpk2* mutant in pairs. Total proteins were isolated from the resulting transformants and incubated with the anti-S-Tag Antibody Agarose beads (Bethyl Laboratories). Proteins bound to anti-S-tag agarose were eluted and used for western blot analysis [13, 31]. The presence of related fusion proteins was detected with the anti-FLAG (Sigma-Aldrich) or anti-S (Abcam) antibody as described [31].

### Spontaneous suppressors of the *cpkA cpk2* mutant

Fast-growing sectors of the *cpkA cpk2* mutant were transferred with sterile toothpicks to fresh oatmeal agar plates. After single-spore isolation, each subculture of spontaneous suppressors was assayed for defects in growth, conidiation, and plant infection [71, 72]. To identify suppressor mutations, all the candidate downstream target genes of PKA were amplified with primers listed in S5 Table and sequenced. Mutation sites were identified by sequence alignment with sequences of target genes in the reference genome [1] and their PCR products.

### Generation of the *cpkA cpk2 Mosfl1* and *cpkA cpk2 MoSFL1*^ΔCT^ mutants

To generate the *cpkA cpk2 Mosfl1* mutant, the upstream and downstream flanking sequences of *MoSFL1* were amplified with primer pairs Sfl1ko1F/Sfl1ko2R and Sfl1ko3F/ Sfl1ko4R (S5 Table), respectively, and fused with the *ble* cassette amplified from pFL6 [73] by double-joint PCR [74]. To generate the *cpkA cpk2 MoSFL1*^ΔCT^ mutant, the flanking sequences of *MoSFL1* were amplified with primer pairs CCSko1F/CCSko2R (S5 Table) and Sfl1ko3F/ Sfl1ko4R (S5 Table). The resulting *MoSFL1* and *MoSFL1*^CT^ gene replacement PCR products were transformed into protoplasts of the *cpkA cpk2* mutant. Putative *Mosfl1* or *MoSFL1*^CT^ mutants were screened by PCR analysis and verified for the deletion of *MoSFL1* or its C-terminal region (496–588 aa).

### Affinity purification and mass spectrometry analysis

The full length *MoSFL1* and *MoSFL1*^**Δ**CT^ fragments were amplified with primers listed in S5 Table and cloned into vector pFL6 by yeast gap repair [70]. The 3×FLAG-*MoSFL1* and 3×FLAG-*MoSFL1*^**Δ**CT^ fusion constructs were rescued from the resulting yeast transformants and transformed into the *cpkA cpk2* mutant. Hyphae of the 3×FLAG-*MoSFL1* and 3×FLAG-*MoSFL1*^**Δ**CT^ transformants were homogenized with a glass beater at 4°C for protein extraction [31, 68]. Proteins eluted from anti-FLAG resins (Sigma-Aldrich) were digested with trypsin and the resulting tryptic peptides were analyzed with nanoflow liquid chromatography tandem mass spectrometry (MS) as described [31, 75–77]. Proteins were identified by searching MS data against NCBI non-redundant *F*. *graminearum* protein database with the SEQUEST^™^ algorithm [78]. At least three independent biological replicates were analyzed to identify proteins that interact with MoSfl1^WT^ and MoSfl1^**Δ**CT^.

### Generation of the *MoSFL1*^S211D^, *MoSFL1*^S211A^, *MoSFL1*^T441D^, *MoSFL1*^T441A^, *MoSFL1*^S554D^, and *MoSFL1*^S554A^ mutant alleles and transformants

To generated the *MoSFL1*^S211D^ allele, PCR fragments amplified with primer pairs MoSfl1-FL5F/MoSfl1-S211D1R and MoSfl1-S211D2F/MoSfl1-FL5R (S5 Table) were connected by overlapping PCR [79] and cloned into vector pFL5 [70] by yeast gap repair. The *MoSFL1*^S211D^ construct was rescued from Trp^+^ yeast transformants and verified for the S211D mutation by sequencing analysis. Similar approaches were used to generate the *MoSFL1*^S211A^, *MoSFL1*^T441D^, *MoSFL1*^T441A^, *MoSFL1*^S554D^, and *MoSFL1*^S554A^ constructs. All the *MoSFL1* mutation alleles were transformed into the *cpkA cpk2* deletion mutant. The resulting transformants were characterized for defects in growth, conidiation, appressorium formation, and plant infection as described [71].

## Supporting information

S1 Fig Domain structures of CpkA and Cpk2 and gene replacement of *CPKA* and *CPK2*. **A**. Domain structures of CpkA and Cpk2. **B**. The *CPKA* and *CPK2* gene replacement constructs were constructed by amplifying the flanking sequences with labelled primers and ligated with the G418 and hygromycin (*hph*) resistance cassettes, respectively. Probe 1 and probe 2 were fragments amplified with labelled primers used for Southern blot hybridization. K, *Kpn*I; N, *Nco*I. **C**. Southern blot analysis with the wild type strain Guy11 and *cpkA cpk2* mutant CAC2. The *cpkA cpk2* mutant had a 4.0-kb *Kpn*I band hybridized with probe 1 (left) and a 2.6-kb *Nco*I band hybridized with probe 2 (right).
(TIF)

S2 Fig Assays for the interaction of Sum1 with CpkA and Cpk2. **A**. Transformant KAS5 expressing the *SUM1*-S and *CPKA*-3×Flag constructs. **B**. Transformant KZS2 expressing the *SUM1*-S and *CPK2*-3×Flag constructs. Western blots of total proteins (Input) and proteins eluted from anti-S-Tag agarose beads (IP) were detected with anti-S, anti-Flag, or anti-actin antibody. Proteins isolated from Guy11 were included as the control. In strains KAS5 and KZS2, the CpkA- and Cpk2-3×FLAG bands were detected in elusions from anti-S beads, indicating that both of them interact with Sum1.
(TIF)

S3 Fig Appressorium formation assays. Conidia of Guy11 and the *cpkA cpk2* mutant were used to inoculate barley (upper panel) and rice (lower panel) leaves. Appressorium formation was assayed 1 dpi. Scale bar = 10 μm.
(TIF)

S4 Fig Assay of growth rate and conidiation of the spontaneous suppressors. Growth rate (**A**) and conidiation (**B**) of Guy11, *cpkA cpk2* mutant, and 20 spontaneous suppressors (CCS1-CCS20) were measured with 7-day-old oatmeal agar cultures. Mean and standard errors were estimated with data from three independent measurements.
(TIF)

S5 Fig Gene replacement of *MoSFL1* and *MoSFL*^CT^. **A**. The *MoSFL1* genomic region, gene-replacement constructs of *MoSFL1* and *MoSFL*^CT^, and PCR primers used. **B**. DNA gel blot analysis with the *cpkA cpk2* (CAC2), *cpkA cpk2 Mosfl1* (TKO4), and *cpkA cpk2 Mosfl1*^CT^ (CTD2) mutants. DNA samples were digested with *Eco*RI and hybridized with probe 1.
(TIF)

S6 Fig Sequence alignment of C-terminal (CT) region of *MoSFL1* with its orthologs from *Fusarium graminearum* (Fg), *Neurospora crassa* (Nc), *Sclerotinia sclerotiorum* (Sc), *Botrytis cinerea* (Bc), *Aspergillus nidulans* (An), *Mocrosporum canis* (Mc), and *Trichophyton rubrum* (Tr). (TIF)

S1 Table Putative Sum1-interacting genes identified by affinity purification. (DOCX)

S2 Table Genes selected for sequencing analysis in suppressor strains. (DOCX)

S3 Table Down-regulated genes in the *cpkA cpk2* double mutant. (XLSX)

S4 Table Suppressor mutations identified in the ORF of *FgSFL1*. (DOCX)

S5 Table PCR primers used in this study. (DOCX)
