# Supplementary figures and images for: PKA activity is essential for relieving the suppression of hyphal growth and appressorium formation by MoSfl1 in Magnaporthe oryzae
Source: PLoS Genet. 2017 Aug 14;13(8):e1006954. doi: 10.1371/journal.pgen.1006954 (PMC5570492; doi:10.1371/journal.pgen.1006954)

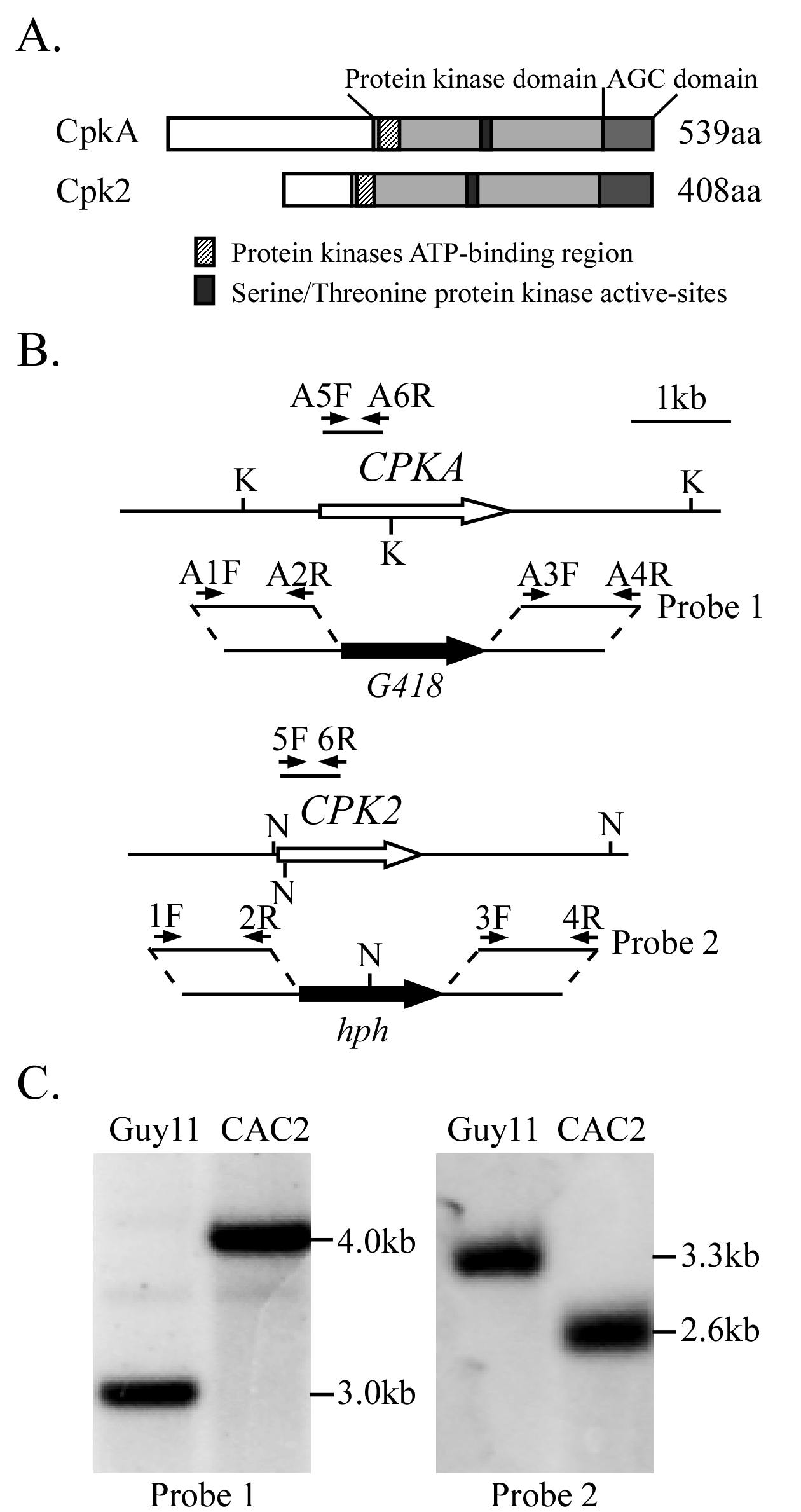

Supplement: S1 Fig — A. Domain structures of CpkA and Cpk2. B. The CPKA and CPK2 gene replacement constructs were constructed by amplifying the flanking sequences with labelled primers and ligated with the G418 and hygromycin (hph) resistance cassettes, respectively. Probe 1 and probe 2 were fragments amplified with labelled primers used for Southern blot hybridization. K, KpnI; N, NcoI. C. Southern blot analysis with the wild type strain Guy11 and cpkA cpk2 mutant CAC2. The cpkA cpk2 mutant had a 4.0-kb KpnI band hybridized with probe 1 (left) and a 2.6-kb NcoI band hybridized with probe 2 (right). (TIF) [file pgen.1006954.s001.tif]

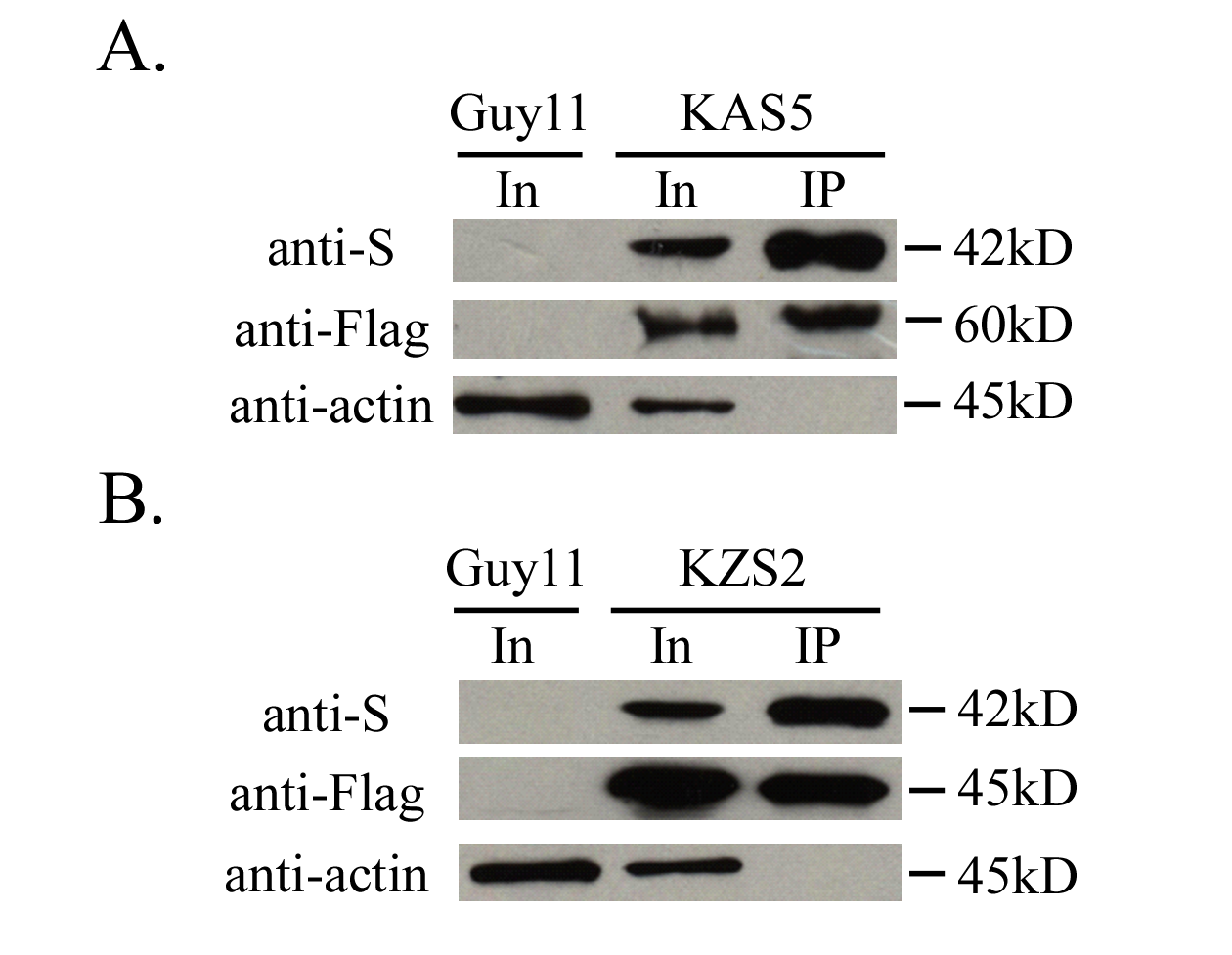

Supplement: S2 Fig — A. Transformant KAS5 expressing the SUM1-S and CPKA-3×Flag constructs. B. Transformant KZS2 expressing the SUM1-S and CPK2-3×Flag constructs. Western blots of total proteins (Input) and proteins eluted from anti-S-Tag agarose beads (IP) were detected with anti-S, anti-Flag, or anti-actin antibody. Proteins isolated from Guy11 were included as the control. In strains KAS5 and KZS2, the CpkA- and Cpk2-3×FLAG bands were detected in elusions from anti-S beads, indicating that both of them interact with Sum1. (TIF) [file pgen.1006954.s002.tif]

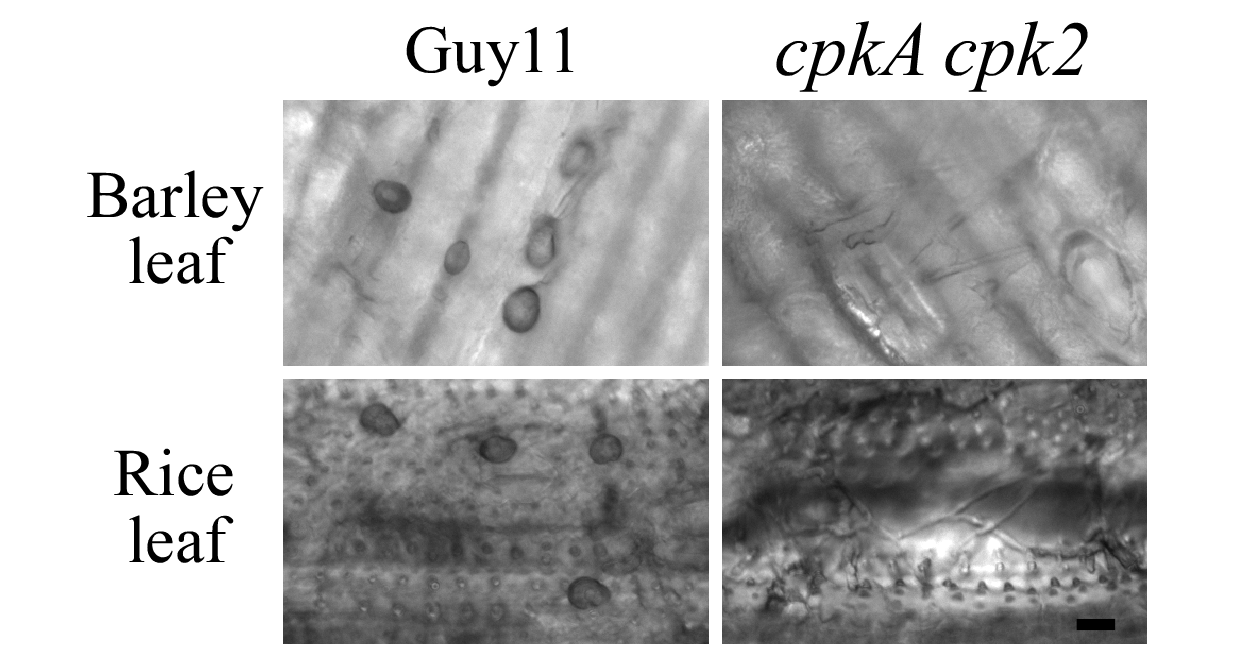

Supplement: S3 Fig — Conidia of Guy11 and the cpkA cpk2 mutant were used to inoculate barley (upper panel) and rice (lower panel) leaves. Appressorium formation was assayed 1 dpi. Scale bar = 10 μm. (TIF) [file pgen.1006954.s003.tif]

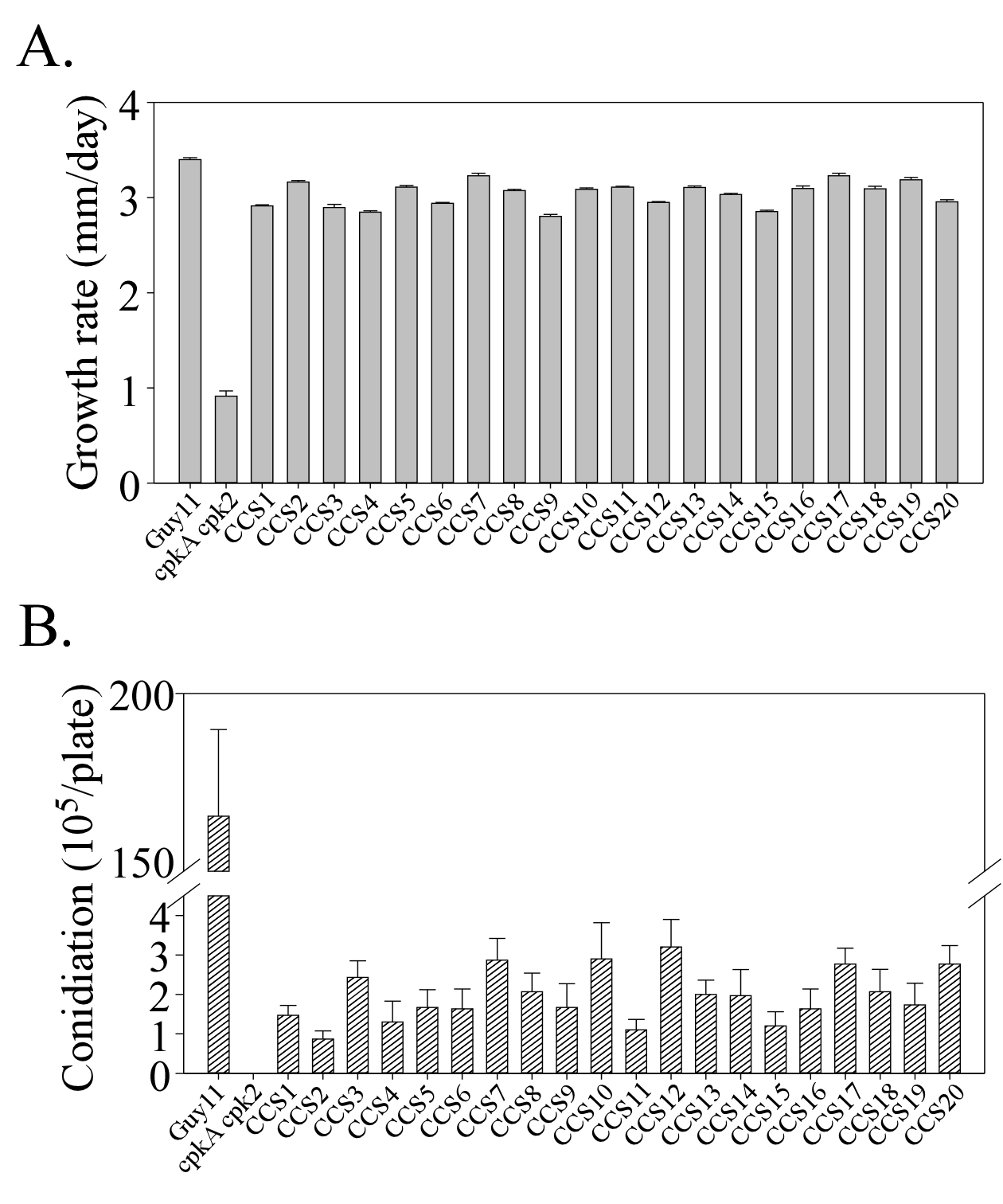

Supplement: S4 Fig — Growth rate (A) and conidiation (B) of Guy11, cpkA cpk2 mutant, and 20 spontaneous suppressors (CCS1-CCS20) were measured with 7-day-old oatmeal agar cultures. Mean and standard errors were estimated with data from three independent measurements. (TIF) [file pgen.1006954.s004.tif]

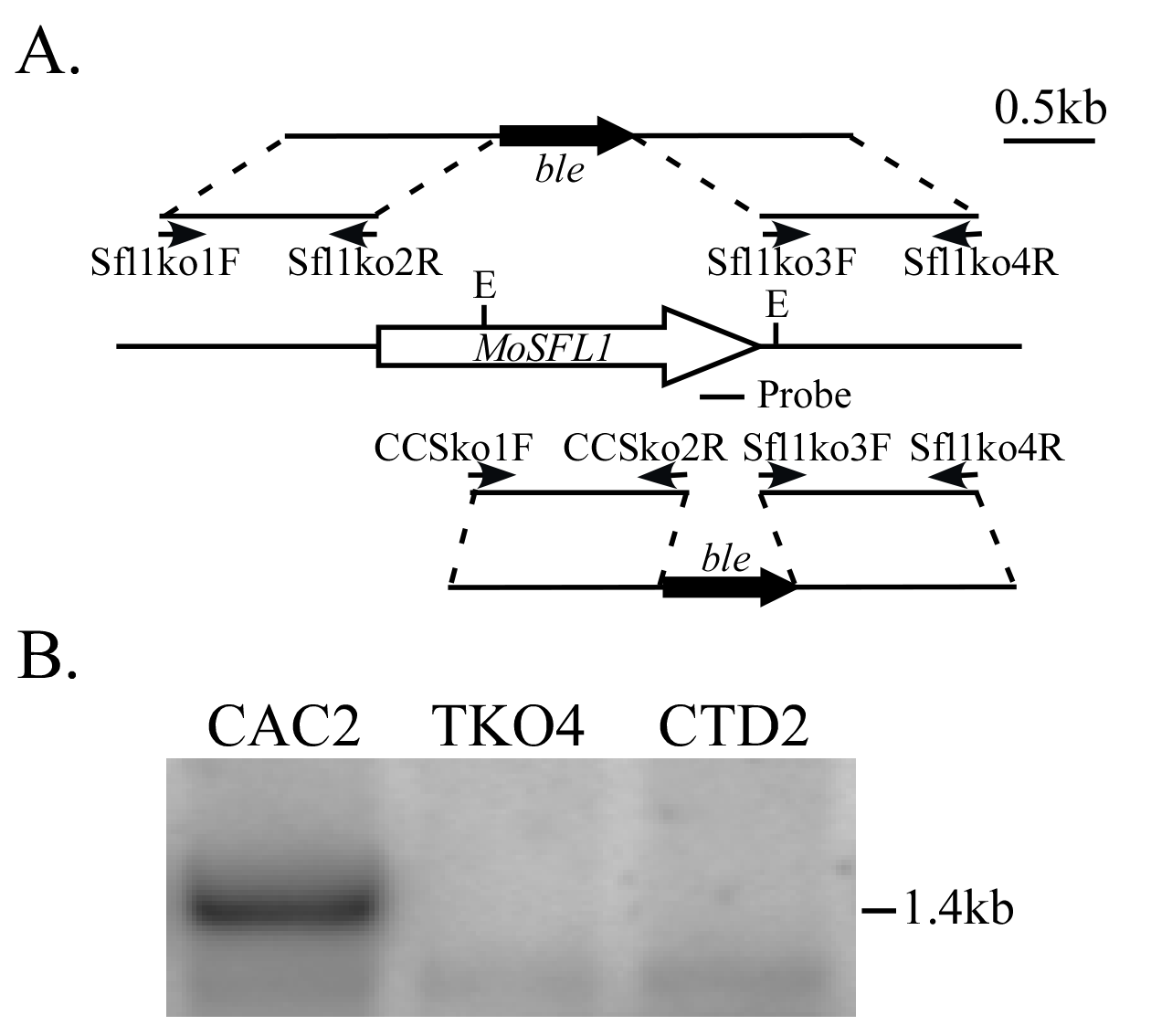

Supplement: S5 Fig — A. The MoSFL1 genomic region, gene-replacement constructs of MoSFL1 and MoSFLCT, and PCR primers used. B. DNA gel blot analysis with the cpkA cpk2 (CAC2), cpkA cpk2 Mosfl1 (TKO4), and cpkA cpk2 Mosfl1CT (CTD2) mutants. DNA samples were digested with EcoRI and hybridized with probe 1. (TIF) [file pgen.1006954.s005.tif]

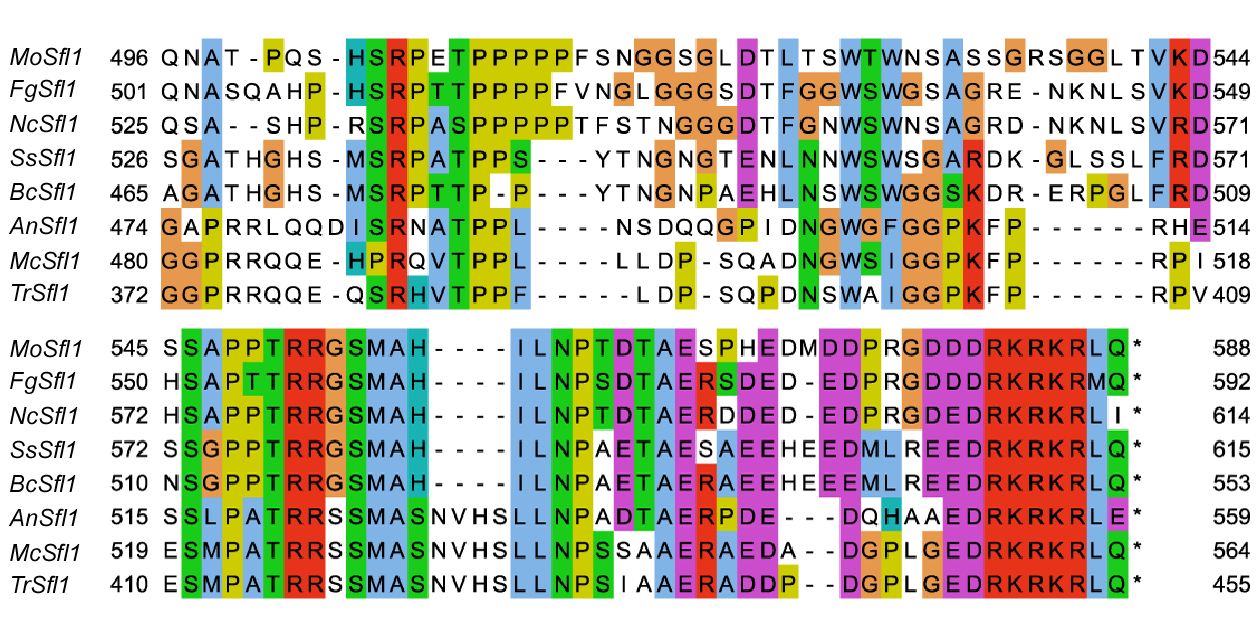

Supplement: S6 Fig — (TIF) [file pgen.1006954.s006.tif]
