# Supplementary material for: PKA activity is essential for relieving the suppression of hyphal growth and appressorium formation by MoSfl1 in Magnaporthe oryzae
Source: PLoS Genet. 2017 Aug 14;13(8):e1006954. doi: 10.1371/journal.pgen.1006954 (PMC5570492; doi:10.1371/journal.pgen.1006954)
Supplement: S1 Table — (DOCX) [file pgen.1006954.s007.docx]

**S1 Table. Putative Sum1-interacting genes identified by affinity purification**

| Genes | Annotation |
| --- | --- |
|  |  |
| MGG_02832 | **conserved hypothetical protein (395 aa)** |
| MGG_06154 | Ras-1 (215 aa) |
| MGG_06368 | **cAMP-dependent protein kinase (540 aa)** |
| MGG_06962 | GTP-binding protein Ypt1 (203 aa) |
| MGG_09952 | GTP-binding nuclear protein Gsp1/Ran (217 aa) |
| MGG_06399 | serine/threonine-protein kinase Ppk15 (913 aa) |
| MGG_09912 | calcium/calmodulin-dependent protein kinase (407 aa) |
| MGG_02829 | casein kinase I (359 aa) |
| MGG_06482 | MAP kinase kinase *MKK1*/*SSP32* (516 aa) |
| MGG_09519 | serine/threonine-protein kinase Cot-1 (421 aa) |
| MGG_08689 | calcium-independent protein kinase C (1161 aa) |
