# Supplementary material for: PKA activity is essential for relieving the suppression of hyphal growth and appressorium formation by MoSfl1 in Magnaporthe oryzae
Source: PLoS Genet. 2017 Aug 14;13(8):e1006954. doi: 10.1371/journal.pgen.1006954 (PMC5570492; doi:10.1371/journal.pgen.1006954)
Supplement: S2 Table — (DOCX) [file pgen.1006954.s008.docx]

**S2 Table. Genes selected for sequencing analysis in suppressor strains**

| Gene | Notes | Reference |
| --- | --- | --- |
| *RIM15* | Deletion of *RIM15* rescues spore viability in the *tpk1* *tpk2* *tpk3* triple mutant | [35] |
| *YAK1* | Overexpression of *YAK1* rescues growth defects of the *tpk1* *tpk2 tpk3* triple mutant | [36] |
| *MSN2* | Deletion of *MSN2* and *MSN4* increases growth in the *tpk2* mutant | [37] |
| *SOK1* | Overexpression of *SOK1* suppresses the growth defect of mutants lacking PKA activity | [38] |
| *SCH9* | Overexpression *SCH9* can functionally rescue a triple TPK deletion mutant | [39] |
| *SFL1* | Downstream target of PKA | [27] |
| *SOM1* | Downstream target of PKA | [40] |
| *CDTF1* | Downstream target of PKA | [40] |
