# Supplementary material for: PKA activity is essential for relieving the suppression of hyphal growth and appressorium formation by MoSfl1 in Magnaporthe oryzae
Source: PLoS Genet. 2017 Aug 14;13(8):e1006954. doi: 10.1371/journal.pgen.1006954 (PMC5570492; doi:10.1371/journal.pgen.1006954)
Supplement: S4 Table — (DOCX) [file pgen.1006954.s010.docx]

**S4 Table. Suppressor mutations identified in the ORF of *FgSFL1***

| **Suppressor strain** | **Mutation** | **Amino acid changes** |
| --- | --- | --- |
| HS1 | C1717 to T | Q501 to stop |
| HS2 | C1717 to T | Q501 to stop |
| HS3 | C1717 to T | Q501 to stop |
| HS4 | C1717 to T | Q501 to stop |
| HS5 | C1717 to T | Q501 to stop |
| HS6 | C1717 to T | Q501 to stop |
| HS7 | G479 to A | W131 to stop |
| HS8 | Insertion of G after A1344 | Frameshift after P378 |
| HS9 | Deletion of TG (757-758) | Frameshift after L197 |
| HS10 | G1195 to T | G328 to stop |
| HS11 | G1195 to T | G328 to stop |
| HS12 | C1717 to T | Q501 to stop |
| HS13 | Deletion of TG (757-758) | Frameshift after L197 |
| HS14 | G451 to A | D132 to N |
| HS15 | C1717 to T | Q501 to stop |
| HS16 | G1598 to A | W461 to stop |
| HS17 | C1717 to T | Q501 to stop |
| HS18 | C1717 to T | Q501 to stop |
| HS19 | C1717 to T | Q501 to stop |
| HS20 | Insertion of A after G1219 | Frameshift after D335 |
| HS21 | Deletion of TG (757-758) | Frameshift after L197 |
| HS22 | C840 to A | H225N |
| HS23 | C1717 to T | Q501 to stop |
| HS24 | Insertion of G after G903 | Frameshift after P245 |
| HS25 | C1717 to T | Q501 to stop |
| HS26 | C1717 to T | Q501 to stop |
| HS27 | Deletion of C1506 | Frameshift after P432 |
| HS28 | Deletion of A742 | Frameshift after G191 |
| HS29 | No mutation identified | No mutation identified |
| HS30 | C1501 to T | Q501 to stop |
