# Supplementary material for: PKA activity is essential for relieving the suppression of hyphal growth and appressorium formation by MoSfl1 in Magnaporthe oryzae
Source: PLoS Genet. 2017 Aug 14;13(8):e1006954. doi: 10.1371/journal.pgen.1006954 (PMC5570492; doi:10.1371/journal.pgen.1006954)
Supplement: S5 Table — (DOCX) [file pgen.1006954.s011.docx]

S5 Table. PCR primers used in this study

| Name | Sequence (5’-3’) | Applications |
| --- | --- | --- |
| A1F | GTGACACCAACAAGCATCCAACTT | *CPKA* knockout |
| A2R | CAGATACGGCAGAGAAATCGCAACCTCCGAGGCGACAATGGGGATTCCTGC | *CPKA* knockout |
| A3F | GTTTAGATTCCAAGTGTCTACTGCTGGCGAGGCTATGATTTGTATTCCACCGG | *CPKA* knockout |
| A4R | TCGTGCGAAAAAATCCCTCCCCTG | *CPKA* knockout |
| A5F | ACTATTGATCCGCAACAAAGCCTG | *CPKA* knockout |
| A6R | AACTTACCCCCGATTTTCTCAGTA | *CPKA* knockout |
| G418/F | TCATTGTCAGATACGGCAGAGAAA | *CPKA* knockout |
| G418/R | CTCTTAATACATCAGACAGTACATGC | *CPKA* knockout |
| 1F | GAGTCGGGATATGCACCAAGTT | *CPK2* knockout |
| 2R | TTGACCTCCACTAGCTCCAGCCAAGCCGGAAGGAATCATCCCCGACGAA | *CPK2* knockout |
| 3F | GAATAGAGTAGATGCCGACCGCGGGTTGTAAAAGCTCAAGCGGAAATCC | *CPK2* knockout |
| 4R | CTTCTTTTTCCTTGCGGTGGTT | *CPK2* knockout |
| 5F | GAGATTCATCTGCACAAAGCTCAAAT | *CPK2* knockout |
| 6R | TTTTGTTAAACTGCTTTGCCCATTGT | *CPK2* knockout |
| HYG/F | GGCTTGGCTGGAGCTAGTGGAGGTCAA | *CPK2* knockout |
| HYG/R | AACCCGCGGTCGGCATCTACTCTATTC | *CPK2* knockout |
| Sum1-S/F | ACTCACTATAGGGCGAATTGGGTACTCAAATTGGTTGCCCGTGCTGGGGTTTCGGAGC | *SUM1*-S |
| Sum1-S/R | TTCGAATTTAGCAGCAGCGGTTTCTTTGGCAGCCTGTAGTGGATCCATT | *SUM1*-S |
| CpkA/FL7F | CGACTCACTATAGGGCGAATTGGGTACTCAAATTGGAGCCCCTCGTTGTCGACTGCTTGT | *CPKA*-3×FLAG |
| CpkA/FL7F | CTTTATAATCACCGTCATGGTCTTTGTAGTCGAATCCAGGGAACAAATTCCCGTA | *CPKA*-3×FLAG |
| Cpk2/FL7F | CGACTCACTATAGGGCGAATTGGGTACTCAAATTGGGGCCATCTCTCACACCGATTCTAG | *CPK2*-3×FLAG |
| Cpk2/FL7R | CTTTATAATCACCGTCATGGTCTTTGTAGTCAAAGTCCTGAAAGTAGTGGTCGTACT | *CPK2*-3×FLAG |
| Sfl1ko1F | CAGCAGGCCAAGAATTGCAAGC | *MoSFL1* knockout |
| Sfl1ko2R | GTGTTGACCTCCACTAGCTCCAGCCACTTGAGACGTACAACAAAGGTCCACG | *MoSFL1* knockout |
| Sfl1ko3F | ACGAATTGCTTGCAGGCATCTCATACTGCAGTGACAAAAGAGCCAGAT | *MoSFL1* knockout |
| Sfl1ko4R | AACAGCCGCCACAGCCGTAAA | *MoSFL1* knockout |
| CCSko1F | AAGCAGTACCCCAAAGGCCGA | *MoSFL1*^CT^ knockout |
| CCSko2R | GTGTTGACCTCCACTAGCTCCAGCCACTTATCGAGTATGTGGAAAATGAGTCCCTG | *MoSFL1*^CT^ knockout |
| MoSfl1/FL6F | TCGATTACAAGGATGACGATGACAAGGCTACCGCCATCCAAACAGC | 3×FLAG-*MoSFL1* |
| MoSfl1/FL6R | ACACTGTAACCCGCAACGAAGAACTCCTCCCCACTCCTTTGCCTGATCTGTAA | 3×FLAG-*MoSFL1* |
| MoCCS/FL6R | CACTGTAACCCGCAACGAAGAACTCCTCCCTATATCGAGTATGTGGAAAATGAGTCCCTG | 3×FLAG-*MoSFL1*^ΔCT^ |
| MoSfl1/FL5F | CAGATCTTGGCTTTCGTAGGAACCCAATCTTCAGCTACCGCCATCCAAACAGC | *MoSFL1*^S211D^ |
| MoSfl1/FL5R | CTTTATAATCACCGTCATGGTCTTTGTAGTCCTGCAGTCGCTTGCGCTTCCT | *MoSFL1*^S211D^ |
| MoSfl1/S211D1R | TTGTGCACAAGGGCGTGGCGGTCGGCACGGCGCTTGATCTCTCGCAA | *MoSFL1*^S211D^ |
| MoSfl1/S211D2F | GAGAGATCAAGCGCCGTGCCGACCGCCACGCCCTTGTGCACAA | *MoSFL1*^S211D^ |
| MoSfl1/T441D1R | GTGACCGCGTATATCTGCCGAGTCGTGTCGTCGAGCTAAGCTACTTGG | *MoSFL1*^T441D^ |
| MoSfl1/T441D2F | AAGTAGCTTAGCTCGACGACACGACTCGGCAGATATACGCGGTCAC | *MoSFL1*^T441D^ |
| MoSfl1/S554D1R | TCGGGTTAAGTATGTGGGCCATGTCTCCCCTCCTCGTCGGTGGTGCT | *MoSFL1*^S554D^ |
| MoSfl1/S554D2F | GCACCACCGACGAGGAGGGGAGACATGGCCCACATACTTAACCCGA | *MoSFL1*^S554D^ |
| MoSfl1/S211A1R | TTGTGCACAAGGGCGTGGCGTGCGGCACGGCGCTTGATCTCTCGCAA | *MoSFL1*^S211A^ |
| MoSfl1/S211A2F | GAGAGATCAAGCGCCGTGCCGCACGCCACGCCCTTGTGCACAA | *MoSFL1*^S211A^ |
| MoSfl1/T441A1R | GTGACCGCGTATATCTGCCGATGCGTGTCGTCGAGCTAAGCTACTTGG | *MoSFL1*^T441A^ |
| MoSfl1/T441A2F | AAGTAGCTTAGCTCGACGACACGCATCGGCAGATATACGCGGTCAC | *MoSFL1*^T441A^ |
| MoSfl1/S554A1R | TCGGGTTAAGTATGTGGGCCATAGCTCCCCTCCTCGTCGGTGGTGCT | *MoSFL1*^S554A^ |
| MoSfl1/S554A2F | GCACCACCGACGAGGAGGGGAGCTATGGCCCACATACTTAACCCGA | *MoSFL1*^S554A^ |
| Cyc8stag/F | ACTCACTATAGGGCGAATTGGGTACTCAAATTGGTTCTGCTAAGACCTGCACCGAACT | *CYC8*-S |
| Cyc8stsg/R | TTCGAATTTAGCAGCAGCGGTTTCTTTGGAGCTCTCAACCTTGGGACCCAT | *CYC8*-S |
| Cyc8check/F | AAATCCTTGGCAACCTCGAAGACG | *CYC8*-S |
| Cyc8check/R | CAACAGGTTGAGACGGGCTTTGAT | *CYC8*-S |
| RIM15seq1F | GGCTCTGCTTCTACCAGACA | *RIM15* sequence |
| RIM15seq2F | GGAGAAAGCCATGTAAGTTG | *RIM15* sequence |
| RIM15seq3F | AAGCTCGAATTGTCGAACTA | *RIM15* sequence |
| RIM15seq4F | CTGAGTATCTCCGGTCACAC | *RIM15* sequence |
| RIM15seq5R | CTAGAGGAGGAGATTGCACA | *RIM15* sequence |
| RIM15seq6F | AAGGCCTGGCAAACATGCTC | *RIM15* sequence |
| RIM15seq7F | ATACGCTACTTCAAGATGAA | *RIM15* sequence |
| RIM15seq8F | TCTTCCAGCTTTTCACAGCC | *RIM15* sequence |
| RIM15seq9F | AATGGTGCCGATGTTGCCAG | *RIM15* sequence |
| RIM15seq10R | TTAGATAAAGTTCATGGGCT | *RIM15* sequence |
| YAK1seq1 | CTAAGGCGTTGCTGGTGCTG | *YAK1* sequence |
| YAK1seq2R | GGCAAAGATGCTGTCTATGAAT | *YAK1* sequence |
| YAK1seq3 | CTCGACAAGAACGACGATCA | *YAK1* sequence |
| YAK1seq4R | GACTCCATCGGCCGCCAATA | *YAK1* sequence |
| MSN2seq1 | GGGGATGCTAATACCTTTTCAG | *MSN2* sequence |
| MSN2seq2 | TGGTCTAGGTTGGCAGTAGAAC | *MSN2* sequence |
| MSN2seq3 | CCAAGATGGATTCTGCCACCAA | *MSN2* sequence |
| MSN2seq3R | CTCCCTTAGGTCCCGCAAACTCTG | *MSN2* sequence |
| SOK1seq1F | GCCGGTGAAGCTTTACAGTTCTCG | *SOK1* sequence |
| SOK1seq2R | TGCGGTCCTCGTCGAATACA | *SOK1* sequence |
| SOK1seq3R | GAAGGGAAGCCAGAGTGAAGGGAG | *SOK1* sequence |
| Sch9seq1 | AACATCCCGACAGCCCAAGAATAT | *SCH9* sequence |
| Sch9seq2 | CGAGCTGATATCAGGTGGCCCGAG | *SCH9* sequence |
| Sch9seq3 | GTTCGGACAGCAACGTCAAATTCG | *SCH9* sequence |
| Sch9seq4 | CGTGAAACGGACTAGAACAAAATCG | *SCH9* sequence |
| MoSfl1seq1F | AGAATTGCAAGCCCTGTCCG | *SFL1* sequence |
| MoSfl1seq2R | GTGTCCTGCGAGCTAAAGGT | *SFL1* sequence |
| MoSfl1seq3F | TCTGTTGCATGAGACTCGAC | *SFL1* sequence |
| MoSfl1seq4R | GCAAGCCAATGATGTCTCCC | *SFL1* sequence |
| MoSfl1seq5F | TCAGCGACGTCTTCCACAAC | *SFL1* sequence |
| MoSfl1seq6R | CAGTCGCTTGCGCTTCCTGT | *SFL1* sequence |
| MoSfl1seq7R | TCAGGTACTTTGGCATCTCC | *SFL1* sequence |
| Som1seq1 | CTTGCATCCAACCGAGAACACACA | *SOM1* sequence |
| Som1seq2 | GCATCGCATCGCTTCCGGAGTA | *SOM1* sequence |
| Som1seq3 | TTACAGGCTATCGCGAATGCATCC | *SOM1* sequence |
| Som1seq4 | CGCACCGCACTTCTTCAAAGGTA | *SOM1* sequence |
| Som1seq5R | GGGTATGATGAGATTTGTGATGAC | *SOM1* sequence |
| Cdtf1seq1 | TTCGACTAGGAACGAACGAATGAG | *CDTF1* sequence |
| Cdtf1seq2 | GTTCCAAAACCAGGCTATCAACCA | *CDTF1* sequence |
| Cdtf1seq3 | ACCCTCAGGTCATGTTGCTCCTTC | *CDTF1* sequence |
| Cdtf1seq4 | GAACTAGCTCGGAACTTGCCTCAG | *CDTF1* sequence |
| Cdtf1seq5 | ACGCAAGAACAGTTGTCCAAGCTC | *CDTF1* sequence |
| Cdtf1seq6 | CGGAAGAGAACTTTGAGGCAAACA | *CDTF1* sequence |
| Cdtf1seq7R | GATCAAAACATGCTGGTTGCCAAA | *CDTF1* sequence |
